# Comparative transcriptomic and plastid development analysis sheds light on the differential carotenoid accumulation in kiwifruit flesh

**DOI:** 10.3389/fpls.2023.1213086

**Published:** 2023-08-30

**Authors:** Nitisha Bhargava, Charles Ampomah-Dwamena, Charlotte Voogd, Andrew C. Allan

**Affiliations:** ^1^ The New Zealand Institute for Plant and Food Research Limited (Plant & Food Research) Mt Albert, Auckland Mail Centre, Auckland, New Zealand; ^2^ School of Biological Sciences, University of Auckland, Auckland, New Zealand

**Keywords:** carotenoids, kiwifruit, chlorophyll, chloroplasts, chromoplasts, βcarotene, RNA-sequencing

## Abstract

Carotenoids are colorful lipophilic isoprenoids synthesized in all photosynthetic organisms which play roles in plant growth and development and provide numerous health benefits in the human diet (precursor of Vitamin A). The commercially popular kiwifruits are golden yellow-fleshed (*Actinidia chinensis*) and green fleshed (*A. deliciosa*) cultivars which have a high carotenoid concentration. Understanding the molecular mechanisms controlling the synthesis and sequestration of carotenoids in *Actinidia* species is key to increasing nutritional value of this crop via breeding. In this study we analyzed fruit with varying flesh color from three *Actinidia* species; orange-fleshed *A. valvata* (OF), yellow-fleshed *A. polygama* (YF) and green-fleshed *A. arguta* (GF). Microscopic analysis revealed that carotenoids accumulated in a crystalline form in YF and OF chromoplasts, with the size of crystals being bigger in OF compared to YF, which also contained globular substructures in the chromoplast. Metabolic profiles were investigated using ultra-performance liquid chromatography (UPLC), which showed that β-carotene was the predominant carotenoid in the OF and YF species, while lutein was the dominant carotenoid in the GF species. Global changes in gene expression were studied between OF and GF (both tetraploid) species using RNA-sequencing which showed higher expression levels of upstream carotenoid biosynthesis-related genes such as *DXS, PSY, GGPPS, PDS, ZISO*, and *ZDS* in OF species compared to GF. However, low expression of downstream pathway genes was observed in both species. Pathway regulatory genes (*OR* and *OR-*L), plastid morphology related genes (*FIBRILLIN*), chlorophyll degradation genes (*SGR*, *SGR-L, RCCR*, and *NYC1*) were upregulated in OF species compared to GF. This suggests chlorophyll degradation (primarily in the initial ripening stages) is accompanied by increased carotenoid production and localization in orange flesh tissue, a contrast from green flesh tissue. These results suggest a coordinated change in the carotenoid pathway, as well as changes in plastid type, are responsible for an orange phenotype in certain kiwifruit species.

## 1 Introduction

All photosynthetic organisms (plants, cyanobacteria and algae) synthesize a class of colored, lipophilic isoprenoids, called carotenoids, that are categorized into two groups based on their chemical structures; carotenes and xanthophylls (oxygenated derivatives of carotenes) (Milani et al., 2017; Maoka, 2020). Both groups are essential and have functions that facilitate plant growth, development and adaptability. The protection of the photosynthetic apparatus from photo-oxidative damage (termed photoprotection) is the primary protective function of carotenoids as excess light leads to a shift in the captured to utilized energy equilibrium, thereby generating reactive oxygen species (ROS). Oxidative cleavage of carotenoids produces apocarotenoids such as strigolactones and abscisic acid (ABA) respectively, which are phytohormones that regulate plant growth and development (Ruiz-Sola et al., 2014; Jia et al., 2018; Felemban et al., 2019). The diverse range of colors (yellow, orange, and red) exhibited by carotenoids, due to conjugated double bonds and functional groups, confers pigmentation to non-photosynthetic tissues (flower, fruits, and vegetables), and facilitates seed dispersal and pollination by animals (Khoo et al., 2011; Maoka, 2020). Carotenoids have benefits as dietary intake of carotenoids through fruits and vegetables or as supplements is essential.

The carotenoid biosynthesis pathway is well conserved between species. The final concentration of carotenoid in tissue is dependent on their synthesis, degradation, and localization (Ampomah-Dwamena et al., 2012). Genes that are nuclear-encoded are synthesized to proteins in the cytoplasm and transported to chloroplast or chromoplasts by a transit peptide sequence located at the N-terminal of the proteins. These enzymes utilize substrates (isopentenyl diphosphate, IPP and dimethylallyl diphosphate, DMAPP) that are derived from the methylerythritol 4-phosphate (MEP) pathway (in plastids) and mevalonic acid (MVA) pathway (in cytosol) for carotenoid synthesis (McGarvey and Croteau, 1995; Nagata et al., 2002; Rodriguez-Concepcion, 2010). Three molecules of IPP are combined with one molecule of DMAPP to produce geranylgeranyl diphosphate via the enzymatic activity of geranylgeranyl diphosphate synthase (GGPPS) (Meier et al., 2011; Sun et al., 2022). The condensation of two molecules of GGPP produces 15-*cis* -phytoene by phytoene synthase (PSY), a rate-limiting enzyme in carotenogenesis (Nisar et al., 2015). Post-transcriptional and post-translational regulation of PSY by the ORANGE (OR) protein has been elucidated. The OR protein prevents PSY protein aggregation via its holdase chaperone activity thereby stabilizing PSY protein (Park et al., 2016). A change of an Arginine to a Histidine (Arg^108^➔ His) of the OR protein (a naturally occurring mutation) results in an increase in carotenogenesis and chromoplast biogenesis in melon (Chayut et al., 2017; Sun et al., 2020).

Phytoene desaturase (PDS) catalyzes the desaturation of 15-*cis*- phytoene to carotene (Hugueney et al., 1992) which is acted on by ζ-carotene isomerase (ZISO) to produce 9,9’-di-*cis*-ζ-carotene (Chen et al., 2010; Beltrán et al., 2015; Rodrigo et al., 2019). Another desaturation reaction by ζ-carotene desaturase (ZDS) followed by isomerization by carotene isomerase (CRTISO) leads to the production of lycopene (Isaacson et al., 2004). The cyclization of lycopene diverts the pathway into two branches which depends on the addition ionone rings. LCYB adds two β-ionone rings to the lycopene structure, thereby diverting the flux to β-carotene (Bai et al., 2009). The hydroxylation of β-carotene leads to the production of β-cryptoxanthin followed by zeaxanthin (DellaPenna and Pogson, 2006; D’Ambrosio et al., 2011), then zeaxanthin epoxidase (ZEP) produces and antheraxanthin violaxanthin. Violaxanthin de-epoxidase (VDE) reverts the flux back from violaxanthin to zeaxanthin (Jahns et al., 2009). The un-utilized excess light energy for photosynthesis is dissipated as heat by a rapidly induced mechanism called non-photochemical quenching (NPQ) (Derks et al., 2015). In higher plants, the NPQ mechanism is regulated by the activity of a xanthophyll cycle (violaxanthin-antheraxanthin-zeaxanthin cycle; Goss and Lepetit, 2015; Giossi et al., 2020) which facilitates plant adaptability in the changing light conditions. Another branch of carotenoid pathway hydroxylates α-carotene by two heme-containing hydroxylases, cytochrome P450 hydroxylase (CYP97A5 and CYP97C3), resulting in the production of lutein (Kim et al., 2009).

Degradation is another factor determining the final carotenoid concentration in plants. Carotenoid cleavage dioxygenase (CCD) and 9-*cis*-carotenoid dioxygenase (NCED) are responsible for the oxidative cleavage of β-carotene and violaxanthin/neoxanthin to produce strigolactones and abscisic acid (Zhang et al., 2009; Zhou et al., 2019). Localization of carotenoids in plastids also determines carotenoid levels, while the plastid type and their deposition unit (plastoglobules, crystalline, membranous and tubular lipid bodies) influences both concentration and profile (Schweiggert and Carle, 2017; Wen et al., 2020).

Kiwifruit (with commercial cultivars being primarily green *A. chinensis* var. *deliciosa* ‘Hayward’ and yellow *A. chinensis* var. *chinensis*) are popular due to their high nutritional value (Wang et al., 2021). In green fruit, chloroplast associated carotenoids (lutein, violaxanthin, neoxanthin and β-carotene) are predominant across the fruit ripening stage with no change in fruit color from green as the fruit ripens retaining chloroplast during fruit development. In contrast, *A. chinensis* fruit changes color from green to golden-yellow (due to chloroplast to chromoplast transition), although there is no significant increase in carotenoid levels. Therefore, the change in the fruit color in *A. chinensis* can be attributed to the reduction in chlorophyll as the fruit matured which unmasks the color due to carotenoids (McGhie and Ainge, 2002; Nishiyama, 2007; Montefiori et al., 2009). Carotenoid composition of non-commercial species belonging to *Actinidia* genus has also been studied (McGhie and Ainge, 2002; Nishiyama et al., 2005; Latocha et al., 2010; Ampomah-Dwamena et al., 2019).

In this study, we examined fruit from three *Actinidia* species with varying flesh color (green, yellow and orange) to explore the metabolic and transcriptomic changes controlling the differential pigment composition, as well as the changes in plastid morphology during maturation. Our findings provide insights into the molecular mechanisms underlying fruit de-greening and carotenogenesis in *Actinidia* species and suggests future targets for breeding of this colorful trait.

## 2 Experimental methods

### 2.1 Plant material and storage conditions

Fruit samples from three *Actinidia* species were studied. *A. arguta* (green flesh), *A. polygama* (yellow flesh), and *A. valvata* (orange flesh) fruits were grown in a Plant and Food Research Orchard, Motueka, New Zealand. Fruits were collected at four different stages of fruit development (Green stage S1, Breaker stage S2, Color change stage S3 and Mature ripe stage S4). Approximately 30 fruit per sample at each stage were separated into skin and flesh excluding seeds and snap frozen using liquid nitrogen and stored at -80˚C.

### 2.2 Fruit flesh color measurement

Fruit flesh color was measured using a Minolta CR-300 chromameter (Konica Minolta, Mahwah, NJ, USA) by using CIELAB color scales (Pathare et al., 2013). A flat section of mesocarp was used for measuring the flesh color. Ten fruits per developmental stage were used to collect the data.

### 2.3 Carotenoid and chlorophyll extraction

Carotenoid and chlorophyll extraction was performed following Ampomah-Dwamena et al. (2015) with some modifications. Fruit harvested at different developmental stages were separated into skin and flesh (outer pericarp) tissues in liquid nitrogen and ground to powder. Powdered tissues were freeze dried for 24 hours before extraction with acetone. Freeze-dried tissue (50-60 mg) was homogenized in 1 mL of acetone with 0.1% butylated hydroxytoluene (BHT). To prevent chlorophyll degradation due to high acidity in the green *A*. *arguta* samples, 50 mg sodium carbonate was added to 1 mL of acetone + 0.1% BHT (Benlloch-Tinoco et al., 2015). The homogenised sample mixtures were covered with aluminium foil to exclude light and extracted overnight on a rotor (120 rpm), at a room temperature of 17 ˚C. Samples were centrifuged at 20,000 x g, at 10 ˚C for 10 minutes and supernatant collected in fresh tubes. The acetone extracts were flush dried under a stream of nitrogen and the dried extracts redissolved in 300 μL of ethanol and 0.1% BHT. Three biological replicates for each developmental stage were used for pigment analysis (except *A. valvata* S2, where only two replicates were used due to lack of tissue availability).

### 2.4 UPLC analysis

Carotenoids and chlorophyll were separated and quantified using Waters ACQUITY H-Class PLUS ultra-performance liquid chromatography (UPLC) system (Waters, Mississauga, Ontario, Canada). The analysis was conducted using ACQUITY UPLC BEH C18 (100 × 2.1 mm, 1.7μm) column and ACQUITY PDA detector for peak detection. The temperature of the column was maintained at 25˚C. A two solvent system was used, solvent A (0.1% formic acid in MilliQ^®^ water) and solvent B (0.1% formic acid in acetonitrile). The solvent elution rate was 0.4 ml min^-1^ with a total analysis time of 17 minutes 50 seconds. The gradient elution profile was as follows: 70% A/30% B (0-4 min), 40% A/60% B (4-6 min), 5% A/95% B (6-10 min), 0% A/100% B (10.5- 15 min), 70% A/30% B (15.5-17.5 min). An aliquot of 10 μL was injected in the UPLC system for carotenoids and chlorophylls peaks detection at 410 nm (for pheophytins), 430 nm (chlorophyll a) and 450 nm (carotenoids and chlorophyll b). Carotenoids were determined based on their comparison of retention time and spectral data with commercial standards (β-carotene, chlorophyll a, chlorophyll b, lutein, and zeaxanthin) and published literature (Snyder et al., 2004; Meléndez-Martínez et al., 2007; Kamffer et al., 2010; Fu et al., 2012; Gupta et al., 2015; Zeb and Ullah, 2017; Matsumoto et al., 2021). The concentration of lutein, chlorophyll a and chlorophyll b were determined using the detected peak area (μv*sec) against the standard curve derived from their standards. The standard curve was derived using concentrations (10 μg/mL, 25 μg/mL, 50 μg/mL, 125 μg/mL, 250 μg/mL) of lutein, chlorophyll a and chlorophyll b standards detected at 450nm, 430nm, 450nm respectively, and was obtained by triplicate injections. The concentration of other carotenoids was determined as lutein equivalent (μg/g dry weight of tissue DW) and chlorophyll derivatives (pheophytin a and pheophorbide a) were quantified as chlorophyll a (also detected at 410 nm for quantification of their derivatives detected at 410 nm) equivalent (Edelenbos et al., 2001; Molina et al., 2022). All the standards were purchased from Sigma-Aldrich (St Louis, MO, United States). The limit of detection (LOD) and limit of quantification (LOQ) were calculated using the standard error (SE) and slope of the calibration curves (Soares et al., 2019) (**Supplementary File 1**).

### 2.5 RNA isolation and quantification of expression levels of carotenoid pathway genes

The two tetraploid *Actinidia* species (*A. polygama* was not included as it is a diploid) with contrasting phenotypes were selected for transcriptomic analysis to better understand the differences in carotenoid biosynthesis pathway and its differential regulation. Total RNA was extracted from the flesh of *A. arguta* (GF) and *A. valvata* (OF) across the four developmental stages (100-150mg) using the Spectrum Plant Total RNA isolation kit (Sigma-Aldrich). The total RNA extracted from the fruit flesh was used in mRNA library preparation for paired-end sequencing by Illumina NovaSeq PE150 (Illumina, NovogeneAIT Genomics, Singapore) generating 20 million reads per sample (three biological replicates of each fruit ripening stage of the two *Actinidia* species). Three independent biological replicates per developmental stage were used to construct a total of 24 cDNA libraries for analysis. The quality of raw reads was improved by stringent data quality control, trimming of adapter sequences and reads, data filtering and error correction to generate high-quality reads. The high-quality reads obtained were mapped to the annotated and published *A. chinensis* reference genome (Pilkington et al., 2018) using STAR v. 2.6.1d (Dobin et al., 2013). The transcript levels (in FPKM, fragment per kilobase per million) of all carotenoid biosynthesis genes across the four developmental stages were evaluated from the transcriptomic data by counting the number of reads mapped to each gene using HTSeq v.0.9.1 (Anders et al., 2015). Approximately 80-85% of reads were uniquely mapped to the genome in all 24 libraries. The FPKM for each biosynthetic gene was calculated based on the length of the mRNA and the number of reads mapped to the sequenced *A. chinensis* genome (Garber et al., 2011). The quantified FPKM values were depicted as a heatmap.2 using the ‘ggplot2’ program in R for visualization (Wickham, 2009). The read count table generated from HTSeq were used for identifying differentially expressed genes (DEGs) using DES_EQ_2 v.1.12.2 (Love et al., 2014) in the package R. Genes with *p-*values < 0.05 and log2 (fold change) > 2 were considered as statistically significant differentially expressed genes. The significantly expressed DEGs were subjected to GO (Gene Ontology) enrichment analysis in ‘Biological Process (BP)’ using clusterProfiler R package (Yu et al., 2012) with Arabidopsis as the reference database. GO terms *p -*values <0.05 were considered significantly enriched.

### 2.6 Transmission electron microscopy

Transmission electron microscopy was used to visualize the ultrastructure of plastids present in the mature fruit flesh. Fresh flesh (1mm cube) from the mesocarp region was fixed for 3 hours using 2.5% glutaraldehyde and freshly prepared 0.1M Sörensens phosphate buffer and washed using the 0.1M Sörensens phosphate buffer. After washing the tissue was post-fixed using 1% osmium tetroxide and 0.1M Sörensens phosphate buffer for an hour followed by a serial dehydration using increasing concentration of ethanol (30%, 50%, 70%, 90%, 100%). The fixed tissues were infiltrated with 100% epoxy resin. After overnight infiltration, fresh resin was used to embed the tissue in embedding molds and polymerized for two days at 60˚C. Ultrathin (70nm) sections were taken using Leica EM UC6 ultramicrotome (Leica Microsystems, Vienna, Austria) provided with DiATOME diamond knife. The thin sections were placed on charged 200-mesh copper grids and stained with uranyl acetate and lead citrate. Grids were imaged in FEI Tecnai 12 TEM (Thermo Fisher Scientific, Eindhoven, The Netherlands) using Gatan UltraScan 1000 camera. All the images were processed and analysed using Gatan Digital Micrograph software (Gatan Inc. Pleasanton, CA, USA).

### 2.7 Bright-field microscopy

A combination of differential interference contrast (DIC) microscopy (Model: Leica DMR microscope, Leica Microsystems (Schweiz) AF- Heerbrugg, Switzerland; Camera: Jenoptik Gryphax Kapella, Jenoptik, Jena, Germany) and bright-field microscopy (Model: Nikon Eclipse Ni-E, Nikon Corporation, Tokyo, Japan) was used to visualize and characterize the plastid development between three species as the fruit developed. Free hand sections of fresh fruit flesh were cut using razor blades and mounted on glass slides without staining.

### 2.8 Statistical analysis

Correlation relationship between transcript levels derived from RNA-Seq analysis of *A. valvata* (OF) and *A. arguta* (GF) and β-carotene concentration were calculated and presented in Pearson’s correlation co-efficient (*r*).

## 3 Results

### 3.1 Changes in fruit flesh color during ripening

**Figure 1:**
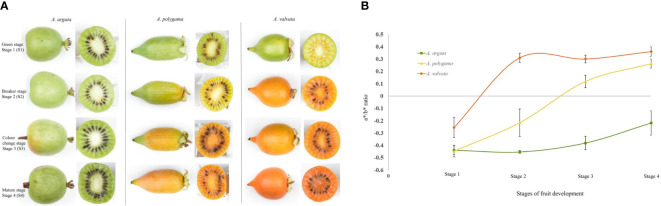
Change in flesh color of (*A*) *arguta, (A) polygama and (A) valvata*. **(A)** Fruit of *Actinidia* species at different ripening stages. **(B)** Chromameter measurements (a*/b* ratio) of flesh. High a*/b* ratio shows the increase in orange color intensity. Error bars represent the standard error of 10 biological replicates per developmental stage.

Fruits of *A. valvata*, *A. polygama*, and *A. arguta* were collected at four different ripening stages i.e., mature green (S1), breaker stage (S2), color change stage (S3) and ripe stage (S4) (**Figure 1A**). These ripening stages occur quickly with each stage being less than 7 days apart. The change in hue angle was measured using Minolta CR300 across the four fruit stages. The three *Actinidia* species had similar green flesh color when harvested. As the fruit reached breaker stage of fruit ripening, the intensity of orange coloration in fruit flesh of *A. valvata* increased such that an increase in the a*/b* ratio was observed (**Figure 1B**). The change from negative to positive a*/b* ratio from S1 to S2 in *A. valvata* underlies the change in color from green to intense yellow at the breaker stage. However, a more gradual change in flesh color was observed during ripening for *A. polygama* with a*/b* ratio ranging from -0.44 to 0.26 coinciding with the four ripening stages. In contrast to the orange and yellow species, the a*/b* ratio of *A. arguta* remained low (-0.43 to -0.21) across all four developmental stages compared to other species, however the a*/b* ratio increased with fruit maturation highlighting the increase in green hue during fruit development.

### 3.2 Plastid development and ultrastructure in *Actinidia* species

To understand pigment variability between the three *Actinidia* species, microscopic analysis was conducted to visualize the type, size, development and ultrastructure of the plastids in the fruit flesh during fruit development. The flesh cells of *Actinidia* fruit were inspected by bright field microscopy during the four ripening stages and the ultrastructure of plastids in the ripe-colored fruit was also observed using transmission electron microscopy (TEM). All the fruit at mature green stage (S1) had chloroplasts as the primary plastid type. As the fruit matured, chromoplasts developed from chloroplasts in the yellow and orange *Actinidia* fruit. However, chloroplasts were the most abundant in green fruit throughout the ripening stages.

**Figure 2:**
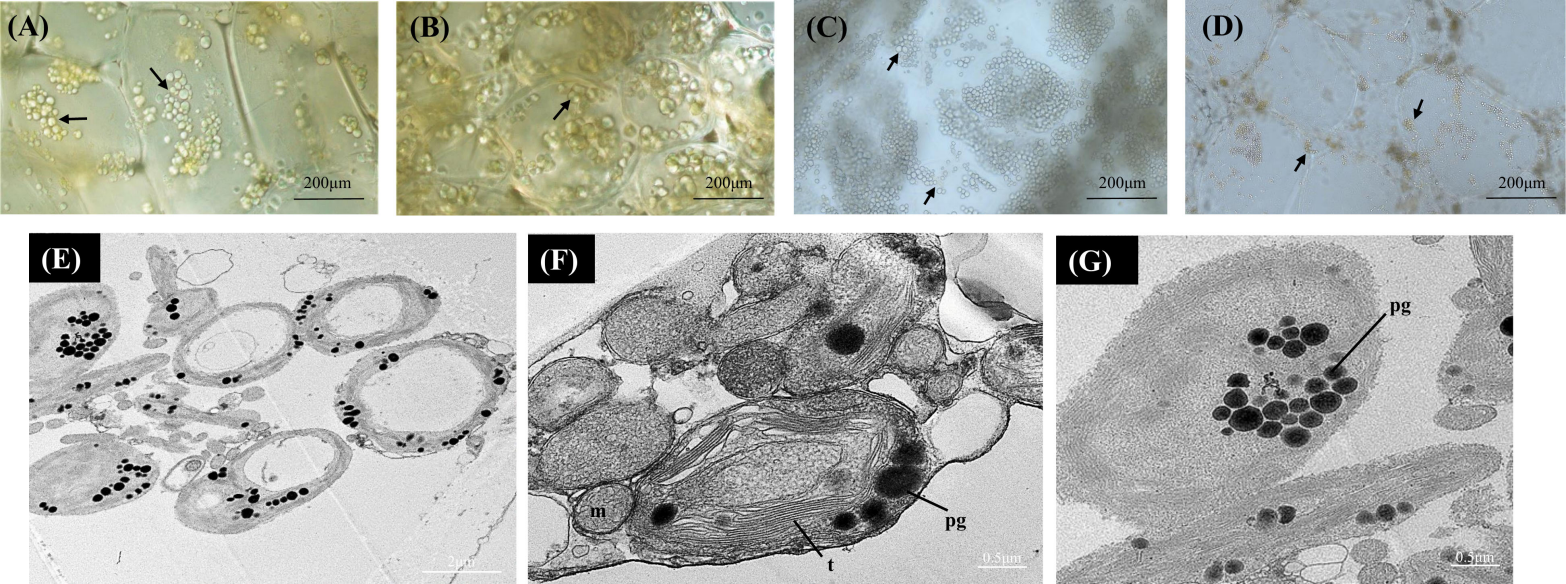
Bright-field micrographs showing plastid development in *Actinidia arguta* flesh across the fruit ripening stages. **(A)** mature-green stage (S1). **(B)** breaker stage (S2). **(C)** color-change stage (S3). **(D)** mature stage (S4). TEM micrographs **(E-G)** of *A. arguta* chloroplasts **(E, F)** and gerontoplasts **(G)**. *arrows* chloroplasts, *m*, mitochondria; *pg*, plastoglobules, and *t*, thylakoids.

In *A. arguta* round green-colored chloroplasts were abundant across the developmental stages under bright field. Actively developing chloroplasts were observed at the earlier ripening stages (S1 and S2) with more yellow-colored plastids observed in the mature fruit (S4) (**Figures 2A-D**). The yellow plastids resembled globular chromoplasts. The ultrastructure of the yellow-colored plastids in S4 was studied using TEM, and the TEM micrographs showed abundance of chloroplasts along with gerontoplasts (plastids developed from chloroplasts during senescence) in the flesh of mature green fruit. The yellow-colored globular plastids observed in the S4 are likely to be senescence associated chloroplasts. Chloroplasts are characterized by intact thylakoids with round electron dense lipid bodies, termed plastoglobules, which also accumulate carotenoids (**Figures 2E-G**). Gerontoplasts, on the other hand, are characterized by degraded thylakoid remnants and enlarged plastoglobules (**Figure 2G**).

**Figure 3:**
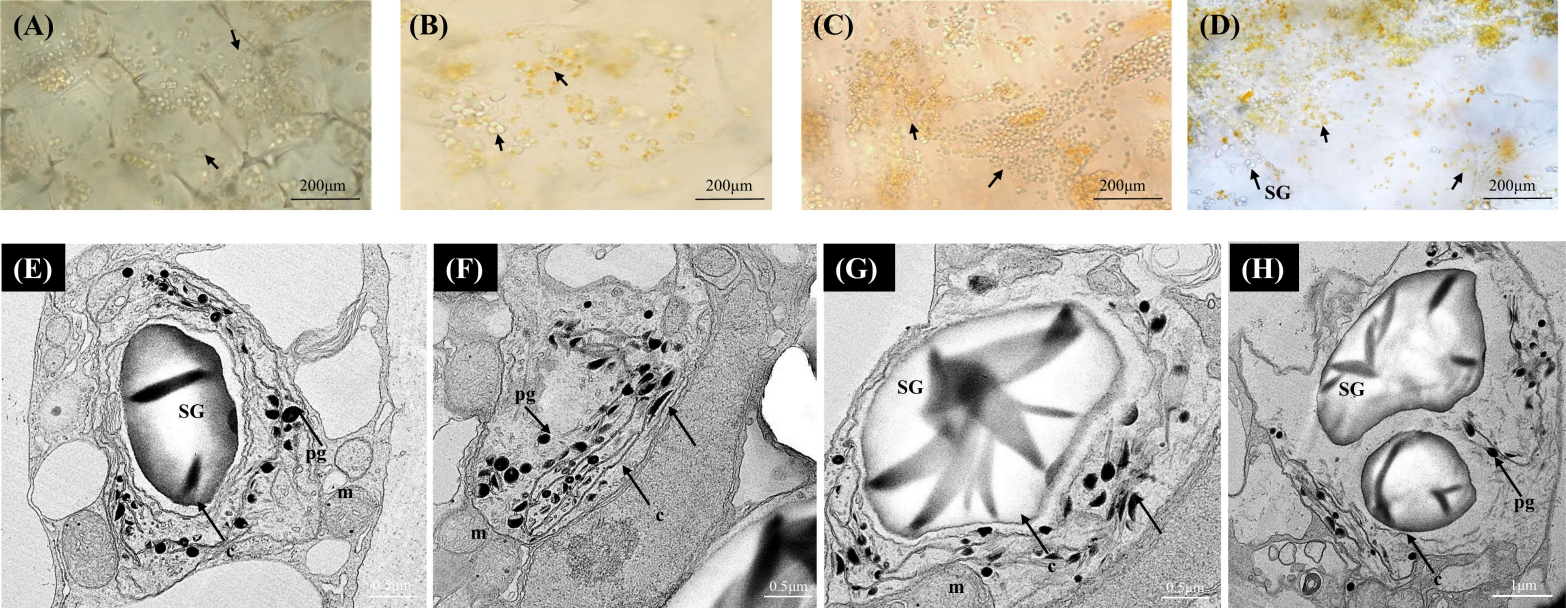
Bright-field micrographs showing plastid development in *Actinidia polygama* flesh across the fruit ripening stages. **(A)** mature-green stage (S1). **(B)** breaker stage (S2). **(C)** color-change stage (S3). **(D)** mature stage (S4). TEM micrographs **(E-H)** of *A. polygama* chromoplasts. *Arrows*; (chloroplasts in **A, B** yellow chromoplasts in **C, D**), *c*, crystal remnants; *m*, mitochondria; *pg*, plastoglobules; *SG*, starch grains, and *t*, thylakoids.

In the yellow *A. polygama* fruit flesh, green chloroplasts were visualized at S1, and at S2 the green chloroplasts appeared to have been converted into yellow chromoplasts (**Figures 3A, B**). As fruit ripening progressed, chloroplast to chromoplast transition increased thereby more immature globular chromoplasts were observed in S3 (**Figure 3C**). In the mature fruit (S4), intensely colored, globular and small crystalloid chromoplasts were predominant (**Figure 3D**). An increase in starch grains was also observed in S4. According to the TEM micrographs (**Figures 3E-H**) of fully mature fruit, chromoplasts were abundant in the flesh with large starch grains within the plastids. Typical large thread-like membranous structures were also visible in the chromoplasts. Round-shaped plastoglobules were also found in abundance in the chromoplasts.

**Figure 4:**
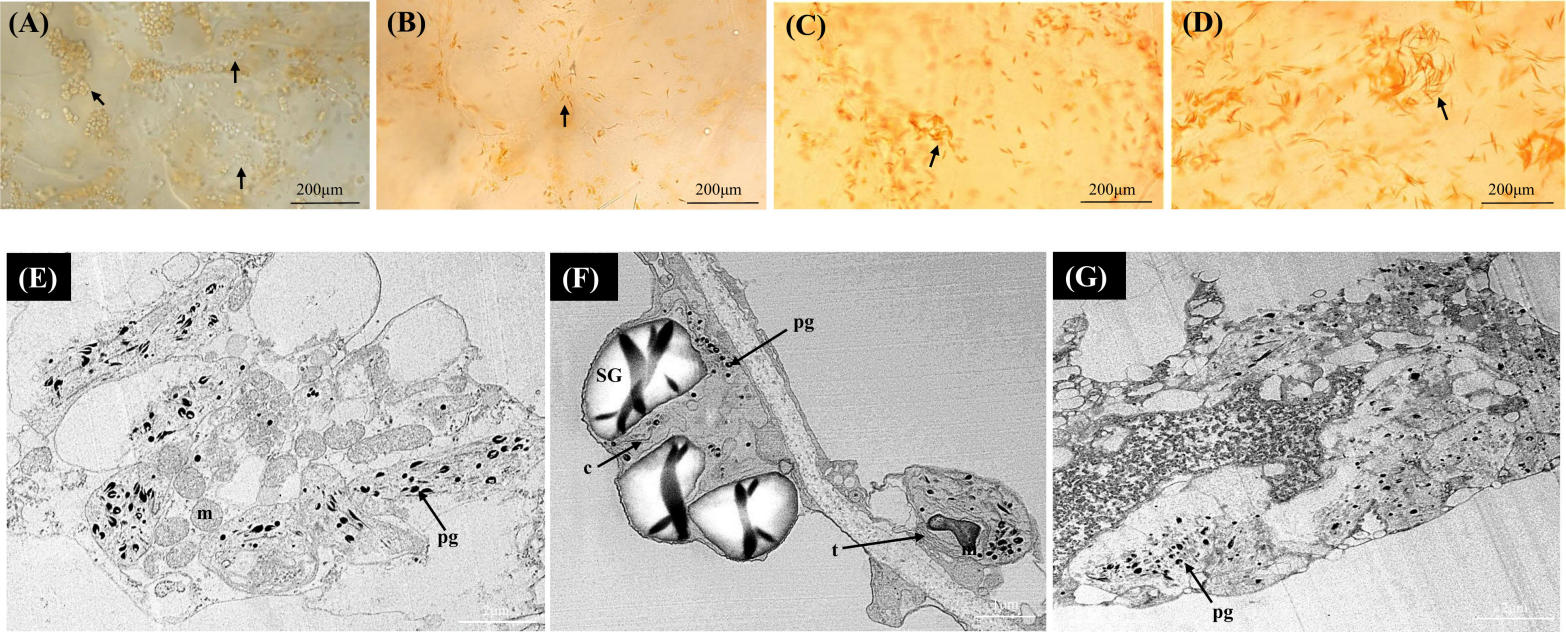
Bright-field micrographs showing plastid development in *Actinidia valvata* flesh across the fruit ripening stages. **(A)** mature-green stage (S1). **(B)** breaker stage (S2). **(C)** color-change stage (S3). **(D)** mature stage (S4). TEM micrographs **(E-G)** of *A. valvata* chromoplasts. *Arrows* (maturing chloroplasts in **A** orange chromoplasts in **B-D**), *c*, crystal remnants; *m*, mitochondria; *pg*, plastoglobules; *SG*, starch grains; and *t*, thylakoids.

In the orange *A. valvata* fruit flesh, chromoplast development was observed at the initial ripening stage (S1) in contrast to the two *Actinidia* fruits where actively developing chromoplasts emerged at breaker stage of fruit development (S2) (**Figure 4A**). Therefore, abundant developing chromoplasts were visualized in S1 and small spindle-shaped chromoplasts emerged from chloroplasts at S2 (**Figures 4A-D**). The size and number of crystalline substructures in the chromoplasts increased as the fruit matured with long crystalline structures observed as a distinctive feature of the orange *Actinidia* fruit. Using TEM (**Figures 4E-G**), chromoplast ultrastructure from S4 revealed numerous electron-dense plastoglobules. In contrast to yellow *Actinidia*, starch grains were sparse. Altogether, differences in the plastid number and ultrastructure were evident among the three species with the orange flesh showing an accelerated chloroplast-to-chromoplast transition and increased plastid concentration. Crystalline chromoplasts were observed in both yellow and orange *Actinidia* fruit with chloroplasts evident in the green *Actinidia* fruit during the ripening.

### 3.3 Changes in carotenoid and chlorophyll content in *Actinidia* fruit flesh and skin

**Figure 5:**
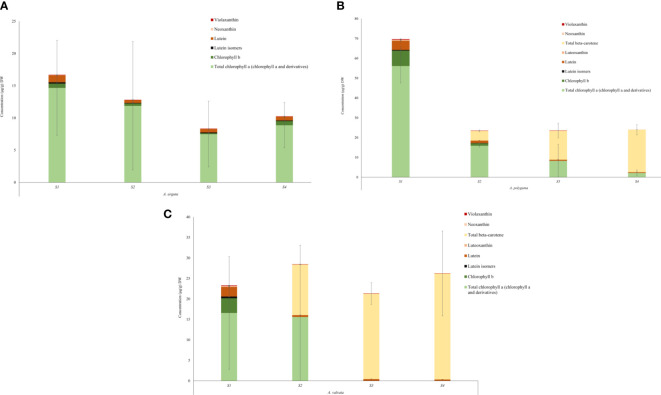
Quantification of chlorophyll and carotenoid content in the flesh of *Actinidia arguta* **(A)**, *A*. *polygama* **(B)**, and *A*. *valvata* **(C)** respectively during the four ripening stages. Carotenoids were measured as lutein equivalents. Chlorophyll a and chlorophyll b were quantified using the standard curve derived from their standards. Total chlorophyll a includes chlorophyll a and its derivatives. Pheophytin a and pheophorbide a (chlorophyll a derivatives) were quantified as chlorophyll a equivalent. The graphs show average of three biological replicates. Error bars are the SD for three biological replicates (except *A*. *valvata* S2 which had only two replicates due to lack of tissue availability).

Metabolic profiles were investigated using ultra-performance liquid chromatography (UPLC) to understand the cause of the varying fruit pigmentation (**Figure 5**). Carotenoids and chlorophyll were extracted from the flesh of the ripening fruits. The total carotenoid concentration differed in the flesh between the three *Actinidia* species, however, the carotenoid profiles were similar between yellow and orange *Actinidia* species. In the *A. arguta*, lutein (ranging from 1.08 μg/g DW– 0.59 μg/g DW) along with traces of lutein isomers, neoxanthin and violaxanthin were observed across the four ripening stages, accounting for the carotenoid profile in the green flesh (**Figure 5A**; **Supplementary File 1**; **Table 1**). The green *A. arguta* fruit could be expected to accumulate chlorophyll a and chlorophyll b during early stages of fruit maturation. Chlorophyll a and chlorophyll b were detected during the four ripening stages with high accumulation of total chlorophyll a (chlorophyll a and its derivatives) (14.67 μg/g DW – 8.88 μg/g DW) compared to chlorophyll b (0.68 μg/g DW - 0.64 μg/g DW).

In the yellow *Actinidia* fruit (**Figure 5B**), the carotenoid profile at S1 stage was similar to the green *A. arguta* fruit at S1 stage with chloroplast-associated carotenoid lutein (4.63 μg/g DW) as the pre-dominant carotenoid. Neoxanthin (0.39 μg/g DW), violaxanthin (0.22 μg/g DW) and luteoxanthin (0.11 μg/g DW) were also observed in the S1 stage with gradual decrease in their concentration as the fruit matured. Luteoxanthin was only detected at S1 of the fruit development. This profile changed dramatically as the fruit matured with β-carotene replacing lutein as the pre-dominant carotenoid during fruit maturation (S2, S3, and S4). The concentration of β-carotene increased 3-fold from S2 to S3 of fruit ripening and ~1.5-fold from S3 to S4. Lutein isomers were observed in the initial stages of fruit ripening (S1 and S2) but were not detected in the mature stages. Total chlorophyll a (chlorophyll a and its derivatives) was detected in all four stages of fruit development however the concentration decreased as the fruit matured (56.17 μg/g DW – 2.23 μg/g DW) and chlorophyll b was detected in S1 and S2 of fruit maturation (7.75 μg/g DW and 1.24 μg/g DW). This decrease in chlorophyll concentration was consistent with the degreening observed during fruit maturation.

The carotenoid profile observed for orange *A. valvata* fruit flesh was similar to *A. polygama* (**Figure 5C**). At S1, lutein (2.34 μg/g DW) was the pre-dominant carotenoid found in the flesh along with traces violaxanthin (0.09 μg/g DW), neoxanthin (0.12 μg/g DW), luteoxanthin (0.12 μg/g DW) and lutein isomers (0.39 μg/g DW). As the fruit matured from S1 to S2, β-carotene became the pre-dominant carotenoid with its accumulation increasing ~2-fold as the fruit matures from S2 to S4 and a decrease in lutein concentration was observed. Chlorophyll b (3.60 μg/g DW) and total chlorophyll a (16.6 μg/g DW in S1 and 15.61 μg/g DW in S2) were detected only in the initial ripening stages (S1 and S2) of fruit maturation (**Figure 5C**). Therefore, the difference in the flesh color phenotype could be explained by the changes in carotenoid and chlorophyll concentration during fruit ripening.

### 3.4 Transcript analysis of carotenoid biosynthetic pathway genes during fruit ripening stages of green *A. arguta* and orange *A. valvata*

**Figure 6:**
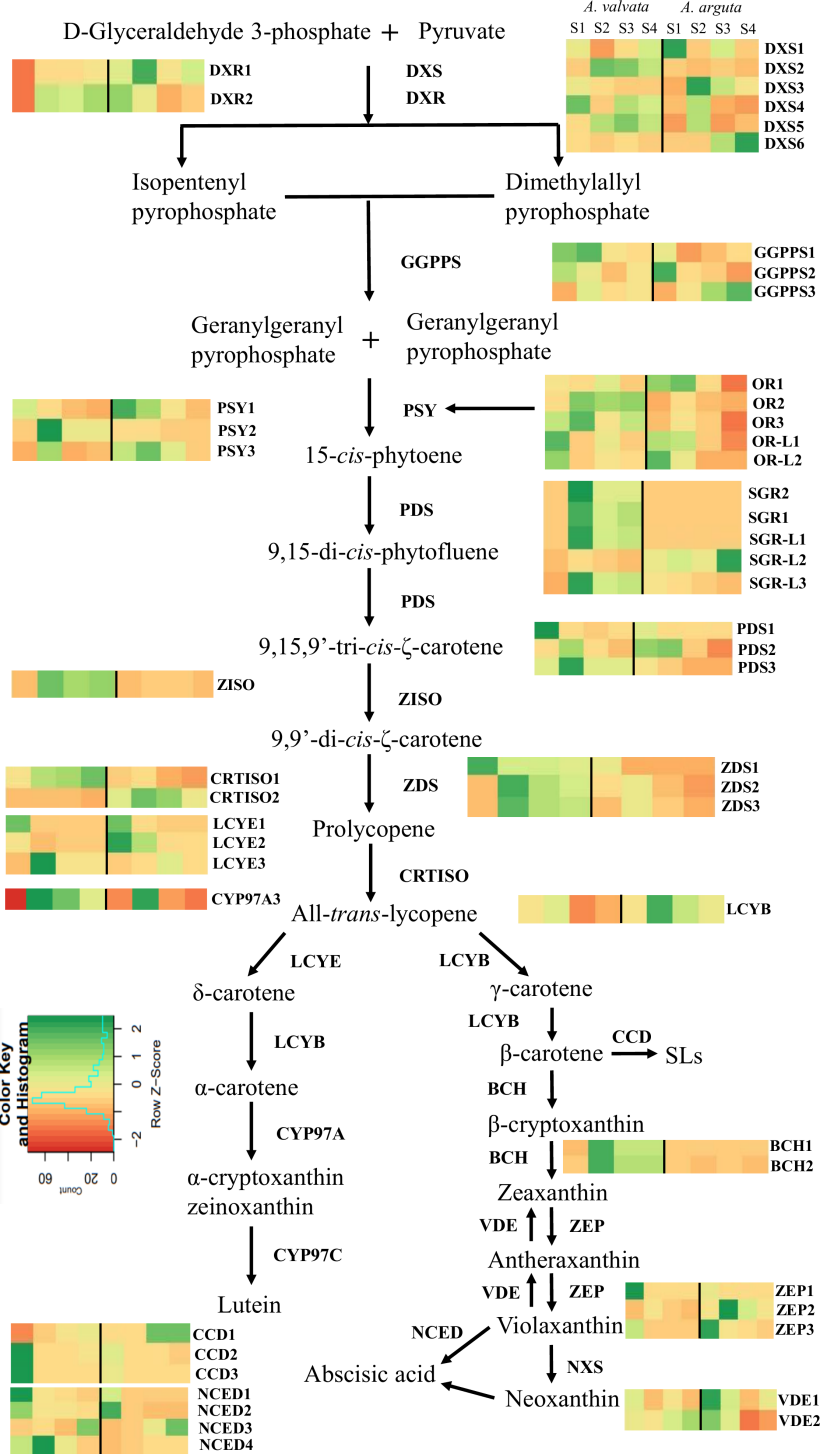
Transcript levels of significantly expressed (p < 0.05) carotenoid pathway genes from RNA-Seq analysis between orange-fleshed *Actinidia valvata* (AV) and green-fleshed *A. arguta* (AA) during four ripening stages (mature green stage, S1; breaker stage, S2; color change stage, S3; mature ripe stage, S4). The FPKM values and the accession numbers are in **Supplementary File 2**, **Table 1**. The heatmap cells from left to right are AV-S1, AV-S2, AV-S3, AV-S4, AA-S1, AA-S2, AA-S3, and AA-S4. The color of the cell represents the FPKM value with green representing high expression levels and red representing low expression levels. DXS, 1-deoxy-D-xylulose-5-phosphate; DXR, 1-deoxy-D-xylulose-5-phosphate reductoisomerase; GGPPS, geranylgeranyl diphosphate synthase; PSY, phytoene synthase; PDS, phytoene desaturase; ZISO, zeta-carotene isomerase; ZDS, zeta-carotene desaturase; CRTISO, carotene isomerase; LCYE, lycopene ϵ-cyclase; CYP97A, β-carotene hydroxylase cytochrome 450 type A; LCYB, lycopene β-cyclase; BCH, β-carotene hydroxylase; ZEP, zeaxanthin epoxidase; VDE, violaxanthin de-epoxidase; NXS, neoxanthin synthase; NCED, 9-*cis*-epoxycarotenoid dioxygenase; CCD, carotenoid cleavage dioxygenase; OR, orange; OR-L, orange-like; SGR, stay green; SGR-L, stay green-like.

To understand the molecular basis of the contrasting phenotypic and metabolic composition between green *A. arguta* and orange *A. valvata* fruits (both tetraploid species), transcriptome profiling was conducted on the fruit flesh at different ripening stages (S1, S2, S3, and S4). A distinct variability between the two species and was observed in PCA analysis (**Supplementary File 2**, **Figure 1**). The transcript levels for the carotenoid biosynthesis pathway genes were expressed in FPKM (**Supplementary File 2**, **Table 1**). Genes with significant differential expression (p < 0.05) were investigated and 44 carotenoid biosynthesis pathway genes were found to be highly expressed including degradation and regulatory genes (**Figure 6**). Among upstream carotenoid pathway genes *DXS2, GGPPS1, GGPPS3, PSY2, PDS3, Z-ISO, ZDS2*, and *ZDS3* showed high transcript accumulation in orange *A. valvata* compared to green *A. arguta* across all four developmental stages. These genes displayed similar expression patterns with a significant increase in the expression levels from S1 (mature green) to S2 (breaker stage) in *A. valvata* fruit following either a subsequent increase or an abrupt decrease in the expression levels in the later stages of the fruit development.

In contrast to the upstream carotenoid pathway genes, the downstream genes showed similar transcript profiles between the two species with low transcript accumulation across the developmental stages, with the exception of *ZEP2* which showed an increase in transcript levels in the green *A. arguta* as the fruit matured compared to *A. valvata*. *BCH1* and *BCH2*, on the other hand, had higher expression in *A. valvata* compared to *A. arguta.* Lycopene beta-cyclase (Acc03917) was differentially expressed (upregulated between S2 and S3), but with low expression levels (**Supplementary File 2**, **Table 1**). LCYE is represented by three gene models which are generally not differentially expressed between the two species (**Figure 6**).

Since the increase in carotenoid concentration in orange *A. valvata* during fruit ripening is also accompanied by de-greening (chlorophyll degradation), expression levels of chlorophyll synthesis and degradation genes were investigated in the transcriptomic data. The genes associated with chlorophyll synthesis and degradation that were significantly expressed (p <0.05) are summarized in (**Table 1**) along with their FPKM values. During the transition from the mature green stage (S1) to the breaker stage (S2) in orange *A. valvata* the genes associated with chlorophyll synthesis are downregulated accompanied by an upregulation in genes associated with chlorophyll degradation. On the other hand, a contrasting gene expression pattern was observed in the green *A. arguta* during fruit ripening. This suggested continuous chlorophyll synthesis during *A. arguta* fruit maturation while in *A. valvata* chlorophyll synthesis decreases from S1 to S2 in orange fruit accompanied by rapid chlorophyll degradation, therefore unmasking the orange pigmentation conferred by carotenoid accumulation.

**Table 1:** Transcript level (FPKM values) of genes associated with chlorophyll synthesis and degradation in *Actinidia valvata* (AV) and *A. arguta* (AA) during fruit development.

Accession number	Gene name	Gene	Function	AV-S1	AV-S2	AV-S3	AV-S4	AA-S1	AA-S2	AA-S3	AA-S4
Acc08661	Chlorophyll synthase	*CLS*	Chlorophyll synthesis	30.2	42.1	52.0	51.2	10.5	6.3	1.4	0.7
Acc06173	Photosystem I chlorophyll a/b binding protein 2	*LHCA2*	Chlorophyll synthesis	32.6	1.0	3.0	2.4	117.5	154.4	35.3	12.0
Acc06486	Chlorophyll b binding protein 3	*LHCII CAB-3*	Chlorophyll synthesis	28.4	0.0	0.2	0.2	62.8	50.5	12.0	7.5
Acc06487	Chlorophyll a-b binding protein 3	*LHCII CAB-3*	Chlorophyll synthesis	54.1	0.0	0.1	0.1	121.6	163.4	36.5	14.3
Acc08260	Chlorophyll a-b binding protein 7	*CAB7*	Chlorophyll synthesis	236.4	27.2	31.2	23.1	574.7	702.7	173.5	57.4
Acc10327	Red chlorophyll catabolite reductase	*RCCR*	Chlorophyll degradation	61.0	181.9	499.4	532.9	36.1	28.8	29.7	35.4
Acc13085	Chlorophyllide b reductase NYC1/Non-yellow coloring 1	*NYC1*	Chlorophyll degradation	11.2	148.5	20.9	17.4	66.9	97.1	69.6	63.2
Acc18283	Chlorophyll a-b binding protein 4	*CAB4*	Chlorophyll synthesis	14.6	0.1	0.1	0.1	98.7	191.0	55.8	17.0
Plastid development											
Acc09726	Fibrillin 1B			689.4	5905.4	12825.1	14465.6	1033.4	1200.9	1109.2	880.2
Acc20301	Fibrillin 11			15.9	20.6	24.2	27.2	27.9	9.7	16.5	11.3
Acc29026	Fibrillin			15.9	22.3	37.6	39.4	7.0	8.6	4.5	4.6
Acc29454	Fibrillin 2			31.9	11.7	19.4	16.4	53.6	43.6	47.0	42.9

There are several enzymes that regulate carotenoid biosynthesis, and then affect carotenoid profile and concentration in plant tissues. The expression levels of regulatory enzymes such as *ORANGE* (*OR*)*, OR-LIKE* (*OR-L*)*, STAY-GREEN* (*SGR*), and *STAY-GREEN LIKE* (*SGR-L*) were investigated in the transcriptomic data (**Figure 6**; **Supplementary File 2**, **Table 1**). The *OR* gene regulates *PSY* post-transcriptionally and post-translationally, thereby stabilizing PSY protein and increasing carotenoid concentration (Park et al., 2016; Sun et al., 2020). The expression of three *OR* genes was detected in the transcriptome with *OR2* exhibiting the highest transcript accumulation in *A. valvata* compared to *A. arguta.* The expression levels increased from S1 to S2 in both species. *FIBRILLIN*, another gene associated with plastid morphology was also investigated (**Table 1**). Significantly high transcript levels were observed in *A. valvata* compared to *A. arguta* (which also accumulated high transcript levels) with a consistent increase in transcript accumulation in *A. valvata* during fruit ripening whereas in *A. arguta* a reverse trend was observed. Due to rapid de-greening during the early stages of *A. valvata* fruit maturation, *STAY-GREEN* (*SGR*) genes associated with the regulation of chlorophyll degradation during senescence were also investigated (Pilkington et al., 2012). Two *STAY-GREEN* genes, *SGR1* and *SGR2* showed a significant increase in expression level in *A. valvata* from the mature green stage (**S1**) to the breaker stage (S2) with an abrupt decrease in later stages of development (**Figure 6**; **Supplementary File 2**, **Table 1**). In contrast, lower transcript levels were detected in *A. arguta* across all four developmental stages. Similarly, two *STAY-GREEN LIKE* genes (*SGR-L1* and *SGR-L3*) showed similar expression patterns as *SGR* genes. Therefore, the increase in the expression levels of upstream carotenoid pathway genes in orange *A. valvata* compared to *A. arguta* and the similar upregulation of enzymes associated with de-greening and carotenoid accumulation in *A. valvata* are consistent with the contrasting metabolic phenotypes of these two *Actinidia* species.

Differential expression analysis was also conducted to identify the differentially expressed genes between the two species across the four developmental stages. A total of 6948 genes were consistently downregulated and 744 genes were upregulated across the four developmental stages between *A. valvata* and *A. arguta* (**Supplementary File 3**). Carotenoid pathway genes such as *DXS2, PSY2, PDS3, BCH1, BCH2, SGR1, SGR2*, *SGR-L1*, and *FIBRILLIN* were identified as upregulated DEGs as they had high expression levels from S2 to S3 in *A. valvata* (**Figure 6**; **Supplementary File 3**). GO enrichment analysis was performed on the upregulated (744) and downregulated (6948) DEGs, focusing the analysis on the Biological Process (BP) domain (**Supplementary File 4**). The analysis revealed significant (*p-*values < 0.05) enrichment of GO terms, with GO terms such as ‘pigment metabolic process’, ‘chloroplast organization’ ‘photosynthesis’, ‘carotenoid metabolic process’, and ‘tetraterpenoid metabolic process’ being enriched for downregulated genes. Similarly, for upregulated DEGs, the GO terms enriched were associated with regulation such as ‘regulation of isoprenoid metabolic process’ and ‘regulation of abscisic acid biosynthetic process’ (**Supplementary File 4**).

To ascertain how transcript abundance of carotenoid pathway genes explain the concentration of β-carotene concentration in *A. valvata*, a Pearson’s correlation analysis was conducted between the expression levels of carotenoid pathway genes, chlorophyll degradation associated genes, *FIBRILLIN* (associated with plastid development) and total β-carotene concentration (**Supplementary File 2**, **Figure 2**; **Supplementary File 2**; **Table 2**). A strong positive correlation was found between *DXR1* and β-carotene concentration (*r =* 0.79). Alternatively, *ZEP2* (*r =* -0.99) and *NCED4* (*r =* -0.97) were negatively correlated with β-carotene concentration. *RCCR*, a gene associated with chlorophyll degradation, has a significant positive correlation with β-carotene concentration (*r* = 0.95, p < 0.05) indicating a decrease in chlorophyll is accompanied by an increase in carotenoid concentration. β-carotene and *FIBRILLIN* displayed a significant positive correlation (*r =* 0.98, p < 0.05).

## 4 Discussion

### 4.1 Chromoplast biogenesis during fruit ripening

Carotenogenesis occurs in pigment-accumulating plastids (chloroplasts and chromoplasts) (Li and Yuan, 2013). Factors that impact the final concentration of carotenoids in any plant tissue are synthesis, degradation and sequestration into these plastids (Ampomah-Dwamena et al., 2012). During fruit ripening, chloroplast-to-chromoplast transition accompanies chlorophyll degradation, dismantling of thylakoids, and an increase in carotenoid accumulation as the fruit matures, thereby influencing their final appearance (Schweiggert et al., 2011; Schaeffer et al., 2017). Chloroplasts are photosynthetically active plastids which synthesize and accumulate xanthophylls such as lutein, violaxanthin, zeaxanthin and antheraxanthin (Joyard et al., 2009). In contrast, chromoplasts are photosynthetically inactive and have larger carotenoid sequestration capacity in plant tissues, therefore, are responsible for the coloration of non-photosynthetic tissues (fruits, vegetables and flowers) (Schweiggert and Carle, 2017; Choi et al., 2021). Several comparative studies, such as in *Capsicum annuum* (Kilcrease et al., 2013), *Physalis* (Wen et al., 2020), watermelon (Zhang et al., 2017; Fang et al., 2020), and papaya (Schweiggert et al., 2011), reveal the diversification of chromoplasts across species is associated with the carotenoid composition and concentration. In this study we aimed to understand the plastid diversity in green-fleshed *A. arguta*, yellow-fleshed *A. polygama*, and orange-fleshed *A. valvata*. Bright-field microscopy was conducted to visualize the plastid development during fruit maturation and TEM of the mature ripe fruit revealed the ultrastructural similarities/differences between the plastids.

During fruit maturation morphological changes in the plastids determine the fruit maturity along with degradation of chlorophyll and increase of carotenoid content. In mature tomato fruit, chromoplasts are found in abundance and are derived from chloroplasts found in the immature green stage of fruit development (Coyago-Cruz et al., 2019). However, in papaya, chromoplasts are derived from proplastids (colorless). Similarly, in sweet oranges, chromoplasts occur due to amyloplast differentiation (Zeng et al., 2015). Chloroplasts were present in the mature green stage (S1) of all the three *Actinidia* species investigated, suggesting a chloroplast to chromoplast transition during fruit ripening. Chloroplasts were retained in green fleshed *A. arguta* fruit throughout the four ripening stages (**Figures 2A-D**). As the fruit ripened, globular, yellow-colored structures were also observed in *A. arguta* fruit along with green chloroplasts. TEM micrographs of the mature fruit flesh suggested the yellow-colored globular structures were senescence associated gerontoplasts plastids derived from chloroplasts (Ling et al., 2021). We found the plastids retained chloroplastic features such as intact thylakoid membranes stacked together into grana along with electron-dense round plastoglobules, that are lipid dense substructures involved in carotenoid sequestration (Brehelin and Kessler, 2008). However, extensive structural changes were observed in gerontoplasts (**Figure 2G**) such as degrading thylakoid membranes and enlarged plastoglobules that accumulate xanthophyll esters (Mulisch and Krupinska, 2013; Sadali et al., 2019).

Yellow-fleshed *A. polygama* and orange-fleshed *A. valvata* showed variability in terms of chromoplast type, size and number. Based on the carotenoid sequestering substructures present in chromoplasts, they can be classified into globular, tubular, crystalline, and reticuloglobular chromoplasts (Egea et al., 2010). Both yellow and orange *Actinidia* fruit had chloroplasts in the green stage (S1) of fruit development. In yellow *A. polygama* fruit, actively dividing chloroplasts coincided with breaker stage (S2) and as the fruit matured, globular and small crystal bodies were observed (**Figures 3A-D**). It should be noted that more than one type of carotenoid sequestering substructure can be found in chromoplasts (Sadali et al., 2019). Globular chromoplasts have plastoglobules as the sequestration substructures (Sadali et al., 2019) and accumulate lutein in yellow *A. chinensis* (Montefiori et al., 2009), β-carotene in mango (Vásquez-Caicedo et al., 2006), β-cryptoxanthin in papaya (Schweiggert et al., 2012). Crystalline chromoplasts accumulate β-carotene crystals in carrots and lycopene in tomato (Kim et al., 2010; Schweiggert et al., 2012; Yazdani et al., 2018). Therefore, presence of globular and crystalline chromoplasts in yellow *A. polygama* fruit suggests the yellow pigmentation could be due to the accumulation of β-carotene or xanthophylls. Similarly, large, orange-colored crystals resembling tomato lycopene crystals (Schweiggert et al., 2012) were observed in orange *A. valvata* fruit. The chloro-to-chromoplast transition was observed in green stage (S1) of the fruit with crystalline substructures found in the breaker (S2) stage coinciding with the dramatic color change (a*/b* ratio). As the fruit matured the size and number of the crystalline chromoplasts increased in later stages of fruit development (**Figures 4A-D**). This suggests pigment intensity varies in correlation with plastid type and size along with the substructures present in the plastids.

### 4.2 Differences in the carotenoid profile and content in Actinidia fruit

The color of the fruit is one of the important agronomic traits which influences consumer preference and is an indicator of fruit maturity. Changes in kiwifruit pigmentation are an indicator of fruit ripening which is associated with degradation of chlorophyll and accumulation of anthocyanins and carotenoids (Montefiori et al., 2009; Montefiori et al., 2009a). The evident change in the fruit color in *A. valvata* and *A. polygama* during fruit ripening was associated with de-greening and increase in carotenoid levels in fruit skin and flesh. Metabolic analysis of the three species showed the orange *A. valvata* fruit accumulated higher levels of carotenoids compared to yellow *A. polygama* fruit. In the mature green stage (S1), chloroplast- associated carotenoids were observed in all the three fruits, with lutein being the pre-dominant carotenoid accumulated in the green stage (**Figure 5**). Total chlorophyll a (chlorophyll a and its derivatives) and chlorophyll b were also detected in the flesh of the three species during the S1 stage. Dramatic changes in pigment profile were observed during S2 stage where a shift in metabolic profile was observed in the yellow *A. polygama* and orange *A. valvata* fruit, with elevation of β-carotene concentration accompanied by the decrease in lutein (McGhie and Ainge, 2002). With advancing ripeness, a large increase in the β-carotene levels was observed in the yellow and orange fruit flesh. Traces of violaxanthin were detected in the all the fruit maturation stages of *A. valvata* and initial ripening stages of *A. polygama* (**Figures 5B, C**). The decrease in violaxanthin and neoxanthin production as the fruit ripened accompanied by the increase in β-carotene in *A. polygama* and *A. valvata* suggests a ‘block’ in the pathway downstream of β-carotene thereby facilitating accumulation of high levels of β-carotene (Sun et al., 2018). Chayut et al. (2017) reported the role of the ‘golden SNP’ (altering arginine to histidine) in *OR* gene inhibits β-carotene turnover thereby creating a ‘block’ in the pathway and increasing β-carotene accumulation in orange melon. The qualitative profile of yellow and orange *Actinidia* fruit was similar, therefore, the difference in the fruit color could be attributed to the differences in the β-carotene concentration which was 1.2-fold higher in orange *A. valvata* fruit compared to yellow *A. polygama* at the ripe stage (S4). Chlorophylls and their derivatives were detected in high concentrations in early stages in *A. valvata* and across the four ripening stages in *A. polygama*. Chlorophyll b is converted to chlorophyll a which is then converted to pheophytin a by a metal chelating substance (MCS) during chlorophyll breakdown (Pilkington et al., 2012). High concentration of total chlorophyll a (chlorophyll and its derivatives) was observed only in the initial ripening stages (S1 and S2) of *A. valvata* fruit (**Figure 5C**), suggesting rapid chlorophyll turnover during initial fruit developmental stages accompanied by a significant increase in carotenoid accumulation, therefore unmasking the carotenoid pigmentation at the early ripening stages in orange *Actinidia* fruit (**Figure 1A**).

The green *A. arguta* fruit accumulated pre-dominantly chloroplast-associated carotenoid, lutein in the flesh with traces of lutein isomers, violaxanthin, and neoxanthin during fruit ripening (**Figure 5A**). Total chlorophyll a (chlorophyll a and derivatives) was found to be the most abundant pigment in green *Actinidia* fruit and were detected during all the four ripening stages (a contrast from orange *Actinidia*) suggesting the presence of chlorophylls and their derivatives contribute to the green flesh phenotype of *A. arguta*. The increase in green hue (**Figure 1B**) in *A. arguta* during fruit maturation could be linked to a high accumulation of chlorophylls and their derivatives for instance, olive-brown colored pigments (pheophytins) or other colorless catabolites (Schelbert et al., 2009; Li et al., 2018). The high accumulation of chlorophyll derivatives (primarily pheophytin a) were present in fairly high concentrations in *Actinidia* fruits and could be linked to the increased in chlorophyll degradation due to low pH conditions during pigment extraction. The variation in carotenoid profile and concentration in *Actinidia* fruit could be linked to differential carotenogenesis and variability in carotenoid sequestration.

### 4.3 Transcriptomic changes reveal the role of upstream carotenoid pathway genes in orange *A. valvata*

A comparative transcriptomic analysis identified key carotenoid pathway genes regulating higher carotenoid levels in orange *A. valvata*. In this study, 44 carotenoid pathway genes were significantly differentially expressed (p <0.05) between the two species with high expression levels. The changes in their expression levels (FPKM) between *A. valvata* and *A. arguta* are summarized in **Figure 6** (**Supplementary File 2**, **Table 1**). The upstream genes which encode enzymes that are responsible for the substrate flux towards the downstream branches showed higher expression levels in orange *A. valvata*. In contrast, the downstream genes exhibited similar expression profiles between *A. valvata* and *A. arguta* across the four developmental stages. High transcript levels (FPKM) for *DXS2, GGPPS1, GGPPS3, PSY2, PDS3, ZISO, ZDS3, BCH1* and *BCH2* (downstream of β-carotene) were quantified in *A. valvata* compared to *A. arguta* across all the four developmental stages with a dramatic increase in transcript levels from green stage (S1) to breaker stage (S2) of the fruit development. The two precursors, i.e. isopentenyl diphosphate (IPP) and dimethylallyl diphosphate (DMAPP), of carotenoid biosynthesis pathway are derived from methylerythritol 4-phosphate (MEP) due to a series of reactions catalysed by 1-deoxy-D-xylulose 5-phosphate synthase (*DXS*) and 1-deoxy-D-xylulose 5-phosphate reductoisomerase (*DXR*) (Rodrίguez-Concepción, 2010). Therefore, these two genes are important in producing the primary substrate for the synthesis of downstream carotenoids. *DXS* catalyzes the rate-limiting step of the MEP pathway and controls carotenoid biosynthesis in tomato (Lois et al., 2000), carrot (Simpson et al., 2016), potato (Henriquez et al., 2016), and rice (You et al., 2020) therefore, influencing carotenoid concentration by providing primary substrate for carotenoid biosynthesis. Three molecules of IPP and one molecule of DMAPP due to geranylgeranyl diphosphate synthase (*GGPPS*) activity produce geranylgeranyl diphosphate (GGPP) which is also a substrate for other isoprenoids (Sun et al., 2018). Overexpression of sweet potato GGPPS in *Arabidopsis thaliana* increased carotenoid concentration in transgenic lines (Chen et al., 2015). Directing the flux (GGPP) towards carotenogenesis is the key known function of *PSY* gene (Camagna et al., 2019). Phytoene synthase (*PSY*) regulates the rate-limiting step of carotenogenesis in most of the plant species and its function in increasing carotenoid levels in tomato (Giorio et al., 2008; Efremov et al., 2020), watermelon (Wu et al., 2020), loquat (Fu et al., 2014), maize (Li et al., 2008), banana (Dhandapani et al., 2017), apple (Ampomah-Dwamena et al., 2015) has been extensively studied. Overexpression of *PSY* gene leads to an increase in carotenoid concentration in several plant species whereas low expression levels result in a decrease in carotenoid content (Efremov et al., 2020). Therefore, the high expression level of *PSY2* in *A. valvata* may account for the high carotenoid levels in the fruit flesh. Similarly, overexpression of *PDS* has been associated with enhanced carotenoid production in tomato (McQuinn et al., 2017) whereas *Arabidopsis pds* mutant produces albino phenotype (Qin et al., 2007). Another, major carotenoid pathway gene that catalyses the hydroxylation of β-carotene are β-carotene hydroxylase (*BCH1* and *BCH2*), resulting in the production of zeaxanthin with β-cryptoxanthin as mono-hydroxylated intermediate (DellaPenna and Pogson, 2006; D’Ambrosio et al., 2011; Song et al., 2021). However, the higher expression level of *BCH1* and *BCH2* in orange *A. valvata* contradicts the high accumulation of β-carotene, thus indicating a more complex regulatory network in *A. valvata* that facilitates high β-carotene accumulation. This large increase in transcript levels of upstream pathway genes from S1 to S2 in *A. valvata* coincided with a significant increase in β-carotene concentration. Thus, the upregulation of upstream pathway genes could be directing the flux towards the synthesis of downstream carotenes and xanthophylls, thereby increasing carotenoid concentration in orange *A. valvata.* Interestingly, 8 DEGs were identified to be involved in carotenoid and chlorophyll biosynthesis pathway. Among these were *DXS2, PSY2, PDS3, BCH1*, and *BCH2* along with chlorophyll degradation genes (*SGR1, SGR2*, and *SGR-L1*) along with plastid development genes, such as *ORANGE (OR2)* and *FIBRILLIN.* GO enrichment analysis revealed significant enrichment of GO terms associated with metabolic processes and their regulation, suggesting the involvement of other genes such as transcription factors (TFs) in the regulation of carotenogenesis in orange *A. valvata*. Although key genes involved in carotenogenesis in *A. valvata* have been identified, their regulation and functionality need to be further investigated and verified.

Correlation analysis was performed between gene expression levels and total β-carotene concentration in *A. valvata* to identify the key pathway genes that may be regulating the orange phenotype (**Supplementary File 2**, **Table 2**). No significant positive correlation (with the exception of *DXR1*) was observed between expression levels of upstream carotenoid pathway genes and β-carotene concentration. High transcript levels of *DXR1* were found in *A. valvata* but the expression levels were higher in *A. arguta*, suggesting increased carotenoid accumulation in *A. valvata* could not be linked to high *DXR1* expression levels. Similarly, no significant negative correlation (except *BCH1* and *SGR1*) was observed between the downstream pathway genes and β-carotene explaining an inverse relationship between the two variables. BCH enzyme catalyses the hydroxylation of β-carotene to zeaxanthin with β-cryptoxanthin as the intermediate. Therefore, a negative correlation explains the potential role of *BCH* in facilitating accumulation of β-carotene in *A. valvata* (Xia et al., 2022). The lack of correlation between many carotenoid biosynthetic gene transcript levels and β-carotene accumulation in *A. valvata* could also be attributed differences in protein sequence between the different species. Therefore, high gene expression of the pathway genes may not be the primary reason contributing to higher carotenoid accumulation in *A. valvata* fruit.

As *A. arguta* retains its green phenotype during fruit ripening whereas in *A. valvata* chlorophyll degradation begins during breaker stage (S2) with a complete loss of chlorophyll in the later stages of fruit ripening, we investigated genes associated with chlorophyll synthesis and degradation in the transcriptomic data (**Table 1**). The transcript levels of genes associated with chlorophyll biosynthesis reduced significantly in orange *A. valvata* fruit during fruit development whereas upregulation of chlorophyll synthesis genes was observed during ripening in *A. arguta* fruit. In contrast to chlorophyll biosynthetic genes, genes associated with chlorophyll degradation red chlorophyll catabolite reductase (*RCCR*), non-yellow coloring 1 (*NYC1*) along with stay-green (*SGR1* and *SGR2*) and stay-green-like (*SGR-L1* and *SGR-L3*) were upregulated in *A. valvata*, while their transcript levels were lower in green *A. arguta*. *SGR* genes have been associated with chlorophyll catabolism in *A. chinensis* (Pilkington et al., 2012) which possesses a yellow phenotype. The SGR protein catabolizes chlorophyll by disrupting the light-harvesting chlorophyll a-b binding complex (LHCP) composed of chlorophyll a-b binding proteins (Park et al., 2007; Peng et al., 2013). Therefore, upregulation of chlorophyll-degrading genes may be regulating the orange phenotype of *A. valvata* as de-greening unmasks the carotenoid pigments in fruit. Alternatively, downregulation of chlorophyll catabolism genes in green *A. arguta* may be delaying the de-greening process during fruit ripening accompanied by downregulation of carotenoid biosynthesis genes. SGR protein has been shown to negatively regulate *PSY* by directly interacting with PSY and inhibiting carotenoid accumulation in tomato (Luo et al., 2013). Pearson’s correlation analysis shows a significant inverse relationship between β-carotene concentration and *SGR1* (*r=* -0.81; *p<* 0.05) transcript levels in *A. valvata*, indicating a potential regulatory role of SGR in β-carotene accumulation (**Supplementary File 2**, **Table 2**).

### 4.4 Role of ORANGE and FIBRILLIN in regulating plastid biogenesis

The differences in metabolic profile and concentration between the three *Actinidia* species can also be attributed to the morphological differences between the plastids, as well as their number. The role of *ORANGE* (*OR*) gene in plastid development has been extensively studied in various model plant systems. OR has a dual function in influencing carotenoid concentration. The OR protein is a DnaJ-like zinc finger protein which is involved in posttranscriptional and posttranslational regulation of PSY protein. It prevents PSY protein misfolding and aggregating, thereby stabilizing PSY protein which in turn leads to an increase in carotenoid concentration (Zhou et al., 2015; Park et al., 2016; Welsch et al., 2018). It has also been associated with expanding sink capacity (Sun et al., 2018). The naturally occurring melon OR-His (with a histidine instead of arginine in the active binding site of the protein) regulates chromoplast size and number by binding to a plastid division factor (ARC3), thus interfering its interaction with another plastid division factor (PARC6), to impair binary fission of chromoplast (Sun et al., 2020). High expression levels of *OR2* gene in *A. valvata* may be responsible for the enlarged crystalline chromoplasts observed during fruit maturation (**Figure 6**; **Supplementary File 2**, **Table 1**). Functional analysis of *OR2* encoded protein can provide further insights into the role of *OR* gene in *Actinidia* genus.

*FIBRILLIN*, a plastoglobulin protein (associated with plastoglobules), increases accumulation of carotenoid pigments due to increase in sink capacity (Kilambi et al., 2013). Chromoplasts in tomato lines overexpressing pepper *FIBRILLIN* retained the thylakoid membranes during fruit ripening but a 2-fold increase in carotenoid concentration was also observed (Pozueta-Romero et al., 1997; Simkin et al., 2007; Kilcrease et al., 2013). *FIBRILLIN* is highly expressed in *A. valvata* during fruit ripening (**Table 1**), and there is a high correlation between the transcript level of *FIBRILLIN* and β-carotene in *A. valvata* (*r =* 0.981, p < 0.05) (**Supplementary File 2**, **Table 2**), suggesting the potential role of *FIBRILLIN* in increasing sink capacity (i.e., formation of lipid structures like plastoglobules and fibrils). A correlation between *FIBRILLIN* transcript levels and carotenoid content (violaxanthin) has been seen previously in *Capsicum annum* fruit (Kilcrease et al., 2015; Watkins and Pogson, 2020).

## 5 Conclusions

Carotenogenesis in many plants has been extensively studied and key pathway genes have been identified. Previous research on kiwifruit has shown similarities in the carotenoid content between green and golden kiwifruit phenotypes. In this study we examined *Actinidia* species with dramatic differences in skin and flesh phenotype. The pre-dominant carotenoid in yellow *A. polygama* and orange *A. valvata* was β-carotene whereas green *A. arguta* accumulated lutein during the fruit ripening. A large change in carotenoid profile was accompanied by early de-greening during ripening in yellow/orange fruit. Differences in plastid type, size and number were observed between the three species. Upregulation of upstream carotenoid pathway genes in orange-flesh fruit (*A. valvata*) compared to green *A. arguta* was accompanied by increased chlorophyll degradation at early ripening stages which facilitates the unmasking of orange flesh color. Future research will focus on transcriptional regulators of key enzymes, such as PSY, and post-transcriptional regulators *OR* and *SGR*, during the dramatic changes that result in the orange-fleshed kiwifruit phenotype.

## Data availability statement

The data presented in the study are deposited in the SRA NCBI repository, accession number PRJNA984935.

## Author contributions

AA, CA-D, and NB designed the research plan. NB designed the experimental plan, performed the experimental work, data analysis and wrote the manuscript with AA and CA-D. NB and CV performed transcriptome analysis. AA and CA-D assisted with data analysis and provided valuable feedback for the manuscript. All authors contributed to the article and approved the submitted version.

## References

[B1000] Ampomah-Dwamena C. Dejnoprat S. Lewis D. Sutherland P. Volz R. K. Allan A. C. (2012). Metabolic and gene expression analysis of apple (Malus× domestica) carotenogenesis. J. Exp. Bot. 63 (12), 4497–4511. 22717407 10.1093/jxb/ers134 PMC3421989

[B2] Ampomah-Dwamena C. Driedonks N. Lewis D. Shumskaya M. Chen X. Y. Wurtzel E. T.. (2015). The Phytoene synthase gene family of apple (Malus x domestica) and its role in controlling fruit carotenoid content. BMC Plant Biol. 15 (1), 185. doi: 10.1186/s12870-015-0573-7 26215656 PMC4517366

[B3] Ampomah-Dwamena C. Thrimawithana A. H. Dejnoprat S. Lewis D. Espley R. V. Allan A. C. (2019). A kiwifruit (Actinidia deliciosa ) R2R3-MYB transcription factor modulates chlorophyll and carotenoid accumulation (Wiley). 10.1111/nph.15362 PMC6585760 30067292

[B4] Anders S. Pyl P. T. Huber W. (2015). HTSeq–a Python framework to work with high-throughput sequencing data. Bioinformatics 31 (2), 166–169. doi: 10.1093/bioinformatics/btu638 25260700 PMC4287950

[B5] Bai L. Kim E. DellaPenna D. Brutnell T. P. (2009). Novel lycopene epsilon cyclase activities in maize revealed through perturbation of carotenoid biosynthesis. Plant J. 59 (4), 588–599. doi: 10.1111/j.1365-313X.2009.03899.x 19392686

[B6] Beltrán J. Kloss B. Hosler J. P. Geng J. Liu A. Modi A.. (2015). Control of carotenoid biosynthesis through a heme-based cis-trans isomerase. Nat. Chem. Biol. 11 (8), 598–605. doi: 10.1038/nchembio.1840 26075523 PMC4509827

[B7] Benlloch-Tinoco M. Kaulmann A. Corte-Real J. Rodrigo D. Martínez-Navarrete N. Bohn T. (2015). Chlorophylls and carotenoids of kiwifruit puree are affected similarly or less by microwave than by conventional heat processing and storage. Food Chem. 187, 254–262. doi: 10.1016/j.foodchem.2015.04.052 25977024

[B9] Bréhélin C. Kessler F. (2008). The plastoglobule: A bag full of lipid biochemistry tricks. Photochem. Photobiol. 84 (6), 1388–1394. doi: 10.1111/j.1751-1097.2008.00459.x 19067960

[B10] Camagna M. Grundmann A. Bär C. Koschmieder J. Beyer P. Welsch R. (2019). Enzyme fusion removes competition for geranylgeranyl diphosphate in carotenogenesis. Plant Physiol. (Bethesda) 179 (3), 1013–1027. doi: 10.1104/pp.18.01026 PMC6393812 30309967

[B11] Chayut N. Yuan H. Ohali S. Meir A. Sa’ar U. Tzuri G.. (2017). Distinct mechanisms of the ORANGE protein in controlling carotenoid flux. Plant Physiol. 173 (1), 376-389. 10.1104/pp.16.01256 PMC5210724 27837090

[B13] Chen W. He S. Liu D. Patil G. B. Zhai H. Wang F.. (2015). A sweetpotato geranylgeranyl pyrophosphate synthase gene, IbGGPS, increases carotenoid content and enhances osmotic stress tolerance in Arabidopsis thaliana. PloS One 10 (9), e0137623. doi: 10.1371/journal.pone.0137623 26376432 PMC4574098

[B12] Chen Y. Li F. Wurtzel E. T. (2010). Isolation and characterization of the Z-ISO gene encoding a missing component of carotenoid biosynthesis in plants. Plant Physiol. (Bethesda) 153 (1), 66–79. doi: 10.1104/pp.110.153916 PMC2862425 20335404

[B14] Choi H. Yi T. Ha S. (2021). Diversity of plastid types and their interconversions. Front. Plant Sci. 12, 692024. doi: 10.3389/fpls.2021.692024 34220916 PMC8248682

[B15] Coyago-Cruz E. Corell M. Moriana A. Mapelli-Brahm P. Hernanz D. Stinco C. M.. (2019). Study of commercial quality parameters, sugars, phenolics, carotenoids and plastids in different tomato varieties. Food Chem. 277, 480–489. doi: 10.1016/j.foodchem.2018.10.139 30502174

[B16] D’Ambrosio D. N. Clugston R. D. Blaner W. S. (2011). Vitamin A metabolism: an update. Nutrients 3 (1), 63–103. doi: 10.3390/nu3010063 21350678 PMC3042718

[B17] DellaPenna D. Pogson B. J. (2006). Vitamin synthesis in plants: tocopherols and carotenoids. Annu. Rev. Plant Biol. 57 (1), 711–738. doi: 10.1146/annurev.arplant.56.032604.144301 16669779

[B18] Derks A. Schaven K. Bruce D. (2015). Diverse mechanisms for photoprotection in photosynthesis. Dynamic regulation of photosystem II excitation in response to rapid environmental change. Biochim. Biophys. Acta (BBA) - Bioenergetics 1847 (4-5), 468–485. 25687894 10.1016/j.bbabio.2015.02.008

[B19] Dhandapani R. Singh V. P. Arora A. Bhattacharya R. C. Rajendran A. (2017). Differential accumulation of β-carotene and tissue specific expression of phytoene synthase (MaPsy) gene in banana (Musa sp) cultivars. J. Food Sci. Technol. 54 (13), 4416–4426. doi: 10.1007/s13197-017-2918-8 29184248 PMC5686022

[B20] Dobin A. Davis C. A. Schlesinger F. Drenkow J. Zaleski C. Jha S.. (2013). STAR: ultrafast universal RNA-seq aligner. Bioinformatics 29 (1), 15–21. doi: 10.1093/bioinformatics/bts635 23104886 PMC3530905

[B21] Edelenbos M. Christensen L. P. Grevsen K. (2001). HPLC determination of chlorophyll and carotenoid pigments in processed green pea cultivars (Pisum sativum L.). J. Agric. Food Chem. 49 (10), 4768–4774. doi: 10.1021/jf010569z 11600019

[B22] Efremov G. I. Slugina M. A. Shchennikova A. V. Kochieva E. Z. (2020). Differential regulation of phytoene synthase PSY1 during fruit carotenogenesis in cultivated and wild tomato species (Solanum section lycopersicon). Plants (Basel) 9 (9), 1169. doi: 10.3390/plants9091169 32916928 PMC7569967

[B23] Egea I. Barsan C. Bian W. Purgatto E. Latché A. Chervin C.. (2010). Chromoplast differentiation: current status and perspectives. Plant Cell Physiol. 51 (10), 1601–1611. doi: 10.1093/pcp/pcq136 20801922

[B24] Fang X. Liu S. Gao P. Liu H. Wang X. Luan F.. (2020). Expression of ClPAP and ClPSY1 in watermelon correlates with chromoplast differentiation, carotenoid accumulation, and flesh color formation. Scientia Hortic. 270, 109437. doi: 10.1016/j.scienta.2020.109437

[B25] Felemban A. Braguy J. Zurbriggen M. D. Al-Babili S. (2019). Apocarotenoids involved in plant development and stress response. Front. Plant Sci. 10, 1168. doi: 10.3389/fpls.2019.01168 31611895 PMC6777418

[B27] Fu X. Feng C. Wang C. Yin X. Lu P. Grierson D.. (2014). Involvement of multiple phytoene synthase genes in tissue- and cultivar-specific accumulation of carotenoids in loquat. J. Exp. Bot. 65 (16), 4679–4689. 24935622 10.1093/jxb/eru257 PMC4115255

[B26] Fu W. Magnúsdóttir M. Brynjólfson S. Palsson B.Ø. Paglia G. (2012). UPLC-UV-MS E analysis for quantification and identification of major carotenoid and chlorophyll species in algae. Anal. Bioanal. Chem. 404, 3145–3154. doi: 10.1007/s00216-012-6434-4 23052878

[B28] Garber M. Grabherr M. G. Guttman M. Trapnell C. (2011). Computational methods for transcriptome annotation and quantification using RNA-seq. Nat. Methods 8 (6), 469–477. doi: 10.1038/nmeth.1613 21623353

[B29] Giorio G. Stigliani A. L. Ambrosio C. (2008). Phytoene synthase genes in tomato (Solanumlycopersicum L.) - new data on the structures, the deduced amino acid sequences and the expression patterns. FEBS J. 275 (3), 527–535. 18167141 10.1111/j.1742-4658.2007.06219.x

[B30] Giossi C. Cartaxana P. Cruz S. (2020). Photoprotective role of neoxanthin in plants and algae. Molecules (Basel Switzerland) 25 (20), 4617. doi: 10.3390/molecules25204617 33050573 PMC7587190

[B31] Goss R. Lepetit B. (2015). Biodiversity of NPQ. J. Plant Physiol. 172, 13–32. doi: 10.1016/j.jplph.2014.03.004 24854581

[B33] Gupta P. Sreelakshmi Y. Sharma R. (2015). A rapid and sensitive method for determination of carotenoids in plant tissues by high performance liquid chromatography. Plant Methods 11 (1), 1–12. doi: 10.1186/s13007-015-0051-0 25688283 PMC4329677

[B34] Henriquez M. A. Soliman A. Li G. Hannoufa A. Ayele B. T. Daayf F. (2016). Molecular cloning, functional characterization and expression of potato (Solanum tuberosum) 1-deoxy-d-xylulose 5-phosphate synthase 1 (StDXS1) in response to Phytophthora infestans. Plant Sci. (Limerick) 243, 71–83. doi: 10.1016/j.plantsci.2015.12.001 26795152

[B35] Hugueney P. Romer S. Kuntz M. Camara B. (1992). Characterization and molecular cloning of a flavoprotein catalyzing the synthesis of phytofluene and zeta-carotene in Capsicum chromoplasts. Eur. J. Biochem. 209 (1), 399–407. doi: 10.1111/j.1432-1033.1992.tb17302.x 1396714

[B36] Isaacson T. Ohad I. Beyer P. Hirschberg J. (2004). Analysis in *vitro* of the enzyme CRTISO establishes a poly-cis-carotenoid biosynthesis pathway in plants. Plant Physiol. 136 (4), 4246–4255. doi: 10.1104/pp.104.052092 15557094 PMC535854

[B37] Jahns P. Latowski D. Strzalka K. (2009). Mechanism and regulation of the violaxanthin cycle: The role of antenna proteins and membrane lipids. Biochim. Biophys. Acta 1787 (1), 3–14. doi: 10.1016/j.bbabio.2008.09.013 18976630

[B38] Jia K. Baz L. Al-Babili S. (2018). From carotenoids to strigolactones. J. Exp. Bot. 69 (9), 2189–2204. doi: 10.1093/jxb/erx476 29253188

[B39] Joyard J. Ferro M. Masselon C. Seigneurin-Berny D. Salvi D. Garin J.. (2009). Chloroplast proteomics and the compartmentation of plastidial isoprenoid biosynthetic pathways. Mol. Plant 2 (6), 1154–1180. doi: 10.1093/mp/ssp088 19969518

[B41] Kamffer Z. Bindon K. A. Oberholster A. (2010). Optimization of a method for the extraction and quantification of carotenoids and chlorophylls during ripening in grape berries (Vitis vinifera cv. Merlot). J. Agric. Food Chem. 58 (11), 6578–6586. doi: 10.1021/jf1004308 20450155

[B40] Khoo H. Prasad K. N. Kong K. Jiang Y. Ismail A. (2011). Carotenoids and their isomers: color pigments in fruits and vegetables. Molecules 16 (2), 1710–1738. doi: 10.3390/molecules16021710 21336241 PMC6259627

[B42] Kilambi H. V. Kumar R. Sharma R. Sreelakshmi Y. (2013). Chromoplast-specific carotenoid-associated protein appears to be important for enhanced accumulation of carotenoids in hp1 tomato fruits. Plant Physiol. (Bethesda) 161 (4), 2085–2101. doi: 10.1104/pp.112.212191 PMC3613478 23400702

[B43] Kilcrease J. Collins A. M. Richins R. D. Timlin J. A. Connell M. A. (2013). Multiple microscopic approaches demonstrate linkage between chromoplast architecture and carotenoid composition in diverse Capsicum annuum fruit. Plant J. 76 (6), 1074–1083. doi: 10.1111/tpj.12351 24118159

[B44] Kilcrease J. Rodriguez-Uribe L. Richins R. D. Arcos J. M. G. Victorino J. O’Connell M. A. (2015). Correlations of carotenoid content and transcript abundances for fibrillin and carotenogenic enzymes in Capsicum annum fruit pericarp. Plant Sci. (Limerick) 232, 57–66. doi: 10.1016/j.plantsci.2014.12.014 25617324

[B46] Kim J. E. Rensing K. H. Douglas C. J. Cheng K. M. (2010). Chromoplasts ultrastructure and estimated carotene content in root secondary phloem of different carrot varieties. Planta 231 (3), 549–558. doi: 10.1007/s00425-009-1071-7 19946704

[B45] Kim J. Smith J. J. Tian L. Della Penna D. (2009). The evolution and function of carotenoid hydroxylases in Arabidopsis. Plant Cell Physiol. 50 (3), 463–479. doi: 10.1093/pcp/pcp005 19147649

[B47] Latocha P. Krupa T. Wo osiak R. Worobiej E. Wilczak J. (2010). Antioxidant activity and chemical difference in fruit of different Actinidia sp. Int. J. Food Sci. Nutr. 61 (4), 381–394. doi: 10.3109/09637480903517788 20113214

[B48] Li F. Vallabhaneni R. Yu J. Rocheford T. Wurtzel E. T. (2008). Maize phytoene synthase gene family: overlapping roles for carotenogenesis in endosperm, photomorphogenesis, and thermal stress tolerance. Plant Physiol. 147 (3), 1334–1346. doi: 10.1104/pp.108.122119 18508954 PMC2442542

[B49] Li L. Yuan H. (2013). Chromoplast biogenesis and carotenoid accumulation. Arch. Biochem. Biophys. 539 (2), 102–109. doi: 10.1016/j.abb.2013.07.002 23851381

[B50] Li X. Zhou R. Xu K. Xu J. Jin J. Fang H.. (2018). Rapid determination of chlorophyll and pheophytin in green tea using fourier transform infrared spectroscopy. Molecules (Basel Switzerland) 23 (5), 1010. doi: 10.3390/molecules23051010 29701638 PMC6100186

[B51] Ling Q. Sadali N. M. Soufi Z. Zhou Y. Huang B. Zeng Y.. (2021). The chloroplast-associated protein degradation pathway controls chromoplast development and fruit ripening in tomato (Springer Science and Business Media LLC). 10.1038/s41477-021-00916-y 34007040

[B52] Lois L. M. Rodríguez-Concepción M. Gallego F. Campos N. Boronat A. (2000). Carotenoid biosynthesis during tomato fruit development: regulatory role of 1-deoxy-D-xylulose 5-phosphate synthase. Plant J. Cell Mol. Biol. 22 (6), 503–513. doi: 10.1046/j.1365-313x.2000.00764.x 10886770

[B53] Love M. I. Huber W. Anders S. (2014). Moderated estimation of fold change and dispersion for RNA-seq data with DESeq2. Genome Biol. 15 (12), 1–21. doi: 10.1186/s13059-014-0550-8 PMC4302049 25516281

[B55] Luo Z. Zhang J. Li J. Yang C. Wang T. Ouyang B.. (2013). A STAY-GREEN protein S l SGR 1 regulates lycopene and β-carotene accumulation by interacting directly with S l PSY 1 during ripening processes in tomato. New Phytol. 198 (2), 442–452. doi: 10.1111/nph.12175 23406468

[B56] Maoka T. (2020). Carotenoids as natural functional pigments. J. Natural Medicines 74 (1), 1–16. doi: 10.1007/s11418-019-01364-x PMC6949322 31588965

[B57] Matsumoto T. Matsuno M. Ikui N. Mizushina Y. Omiya Y. Ishibashi R.. (2021). Identification of pheophorbide a as an inhibitor of receptor for advanced glycation end products in Mallotus japonicus. J. Natural Medicines 75, 675–681. doi: 10.1007/s11418-021-01495-0 33625682

[B58] McGarvey D. J. Croteau R. (1995). Terpenoid metabolism. Plant Cell 7 (7), 1015. 7640522 10.1105/tpc.7.7.1015 PMC160903

[B59] McGhie T. K. Ainge G. D. (2002). Color in fruit of the genus actinidia: Carotenoid and chlorophyll compositions. J. Agric. Food Chem. 50 (1), 117–121. doi: 10.1021/jf010677l 11754554

[B60] McQuinn R. P. Wong B. Giovannoni J. J. (2017). AtPDS overexpression in tomato: exposing unique patterns of carotenoid self-regulation and an alternative strategy for the enhancement of fruit carotenoid content (Wiley). 10.1111/pbi.12789 PMC5787846 28703352

[B61] Meier S. Tzfadia O. Vallabhaneni R. Gehring C. Wurtzel E. T. (2011). A transcriptional analysis of carotenoid, chlorophyll and plastidial isoprenoid biosynthesis genes during development and osmotic stress responses in Arabidopsis thaliana. BMC Syst. Biol. 5 (75), 77. doi: 10.1186/1752-0509-5-77 21595952 PMC3123201

[B62] Meléndez-Martínez A. J. Britton G. Vicario I. M. Heredia F. J. (2007). Relationship between the colour and the chemical structure of carotenoid pigments. Food Chem. 101 (3), 1145–1150. doi: 10.1016/j.foodchem.2006.03.015

[B64] Milani A. Basirnejad M. Shahbazi S. Bolhassani A. (2017). Carotenoids: biochemistry, pharmacology and treatment. Br. J. Pharmacol. 174 (11), 1290–1324. 27638711 10.1111/bph.13625 PMC5429337

[B65] Molina A. K. Gomes L. C. Prieto M. A. Ferreira I. C. Pereira C. Dias M. I.. (2022). Extraction of chlorophylls from Daucus carota L. and Solanum lycopersicum var. cerasiforme crop by-products. Food Chem. Adv. 1, 100048.

[B67] Montefiori M. Comeskey D. J. Wohlers M. McGhie T. K. (2009a). Characterization and quantification of anthocyanins in red kiwifruit (Actinidia spp.). J. Agric. Food Chem. 57 (15), 6856–6861. doi: 10.1021/jf900800z 19572542

[B66] Montefiori M. McGhie T. K. Hallett I. C. Costa G. (2009). Changes in pigments and plastid ultrastructure during ripening of green-fleshed and yellow-fleshed kiwifruit. Scientia Hortic. 119 (4), 377–387. doi: 10.1016/j.scienta.2008.08.022

[B1001] Mulisch M. Krupinska K. (2013). Ultrastructural analyses of senescence associated dismantling of chloroplasts revisited. Plastid development in leaves during growth and senescence, 307–335.

[B68] Nagata N. Suzuki M. Yoshida S. Muranaka T. (2002). Mevalonic acid partially restores chloroplast and etioplast development in Arabidopsis lacking the non-mevalonate pathway. Planta 216 (2), 345–350. doi: 10.1007/s00425-002-0871-9 12447549

[B69] Nisar N. Li L. Lu S. Khin N. Pogson B. (2015). Carotenoid metabolism in plants. Mol. Plant 8 (1), 68–82. doi: 10.1016/j.molp.2014.12.007 25578273

[B70] Nishiyama I. (2007). Fruits of the Actinidia genus. Adv. Food Nutr. Res., 52, 293-324. 10.1016/S1043-4526(06)52006-6 17425948

[B71] Nishiyama I. Fukuda T. Oota T. (2005). Genotypic differences in chlorophyll, lutein, and β-carotene contents in the fruits of actinidia species. J. Agric. Food Chem. 53 (16), 6403–6407. doi: 10.1021/jf050785y 16076125

[B72] Park S. Kim H. S. Jung Y. J. Kim S. H. Ji C. Y. Wang Z.. (2016). Orange protein has a role in phytoene synthase stabilization in sweetpotato (Springer Science and Business Media LLC). 10.1038/srep33563 PMC5025653 27633588

[B74] Park S. Yu J. Park J. Li J. Yoo S. Lee N.. (2007). Senescence-induced staygreen protein regulates chlorophyll degradation. THE Plant Cell 19 (5), 1649–1664. doi: 10.1105/tpc.106.044891 17513504 PMC1913741

[B75] Pathare P. B. Opara U. L. Al-Said F. A. (2013). Colour measurement and analysis in fresh and processed foods: A review. Food bioprocess Technol. 6 (1), 36–60. doi: 10.1007/s11947-012-0867-9

[B76] Peng G. Xie X. Jiang Q. Song S. Xu C. (2013). Chlorophyll a/b binding protein plays a key role in natural and ethylene-induced degreening of Ponkan (Citrus reticulata Blanco). Scientia Hortic. 160, 37–43. doi: 10.1016/j.scienta.2013.05.022

[B78] Pilkington S. M. Crowhurst R. Hilario E. Nardozza S. Fraser L. Peng Y.. (2018). A manually annotated Actinidia chinensis var. chinensis (kiwifruit) genome highlights the challenges associated with draft genomes and gene prediction in plants (Springer Science and Business Media LLC). 10.1186/s12864-018-4656-3 PMC5902842 29661190

[B77] Pilkington S. M. Montefiori M. Jameson P. E. Allan A. C. (2012). The control of chlorophyll levels in maturing kiwifruit. Planta 236 (5), 1615–1628. doi: 10.1007/s00425-012-1723-x 22843245

[B79] Pozueta-Romero J. Rafia F. Houlne G. Cheniclet C. Carde J. P. Schantz M. L.. (1997). A ubiquitous plant housekeeping gene, PAP, encodes a major protein component of bell pepper chromoplasts. Plant Physiol. 115 (3), 1185–1194. doi: 10.1104/pp.115.3.1185 9390444 PMC158583

[B80] Qin G. Gu H. Ma L. Peng Y. Deng X. W. Chen Z.. (2007). Disruption of phytoene desaturase gene results in albino and dwarf phenotypes in Arabidopsis by impairing chlorophyll, carotenoid, and gibberellin biosynthesis. Cell Res. 17 (5), 471–482. doi: 10.1038/cr.2007.40 17486124

[B81] Rodrigo M. J. Lado J. Alós E. Alquézar B. Dery O. Hirschberg J.. (2019). A mutant allele of ζ-carotene isomerase (Z-ISO) is associated with the yellow pigmentation of the “Pinalate” sweet orange mutant and reveals new insights into its role in fruit carotenogenesis (Springer Science and Business Media LLC). 10.1186/s12870-019-2078-2 PMC6829850 31684878

[B82] Rodríguez-Concepción M. (2010). Supply of precursors for carotenoid biosynthesis in plants. Arch. Biochem. Biophys. 504 (1), 118–122. doi: 10.1016/j.abb.2010.06.016 20561506

[B1] Ruiz-Sola M. Á. (2014). A root specific induction of carotenoid biosynthesis contributes to ABA production upon salt stress in arabidopsis 9, 3. doi: 10.1371/journal.pone.0090765 PMC3942475 24595399

[B83] Sadali N. M. Sowden R. G. Ling Q. Jarvis R. P. (2019). Differentiation of chromoplasts and other plastids in plants. Plant Cell Rep. 38 (7), 803–818. doi: 10.1007/s00299-019-02420-2 31079194 PMC6584231

[B84] Schaeffer S. M. Christian R. Castro-Velasquez N. Hyden B. Lynch-Holm V. Dhingra A. (2017). Comparative ultrastructure of fruit plastids in three genetically diverse genotypes of apple (Malus × domestica Borkh.) during development. Plant Cell Rep. 36 (10), 1627–1640. doi: 10.1007/s00299-017-2179-z 28698906 PMC5693628

[B85] Schelbert S. Aubry S. Burla B. Agne B. Kessler F. Krupinska K.. (2009). Pheophytin pheophorbide hydrolase (Pheophytinase) is involved in chlorophyll breakdown during leaf senescence in arabidopsis. THE Plant Cell 21 (3), 767–785. doi: 10.1105/tpc.108.064089 19304936 PMC2671698

[B86] Schweiggert R. M. Carle R. (2017). Carotenoid Deposition in Plant And Animal Foods and Its Impact on Bioavailability (Informa UK Limited). 10.1080/10408398.2015.1012756 26115350

[B87] Schweiggert R. M. Mezger D. Schimpf F. Steingass C. B. Carle R. (2012). Influence of chromoplast morphology on carotenoid bioaccessibility of carrot, mango, papaya, and tomato. Food Chem. 135 (4), 2736–2742. doi: 10.1016/j.foodchem.2012.07.035 22980866

[B88] Schweiggert R. M. Steingass C. B. Heller A. Esquivel P. Carle R. (2011). Characterization of chromoplasts and carotenoids of red- and yellow-fleshed papaya (Carica papaya L.). Planta 234 (5), 1031–1044. doi: 10.1007/s00425-011-1457-1 21706336

[B89] Simkin A. J. Gaffé J. Alcaraz J. Carde J. Bramley P. M. Fraser P. D.. (2007). Fibrillin influence on plastid ultrastructure and pigment content in tomato fruit. Phytochem. (Oxford) 68 (11), 1545–1556. doi: 10.1016/j.phytochem.2007.03.014 17466343

[B90] Simpson K. Quiroz L. F. Rodriguez-Concepción M. Stange C. R. (2016). Differential contribution of the first two enzymes of the MEP pathway to the supply of metabolic precursors for carotenoid and chlorophyll biosynthesis in carrot (Daucus carota). Front. Plant Sci. 7, 1344. doi: 10.3389/fpls.2016.01344 27630663 PMC5005961

[B91] Snyder A. M. Clark B. M. Robert B. Ruban A. V. Bungard R. A. (2004). Carotenoid specificity of light-harvesting complex II binding sites: occurrence of 9-cis-violaxanthin in the neoxanthin-binding site in the parasitic angiosperm Cuscuta reflexa. J. Biol. Chem. 279 (7), 5162–5168. doi: 10.1074/jbc.M309676200 14610095

[B92] Soares A. T. da Costa D. C. Vieira A. A. H. Antoniosi Filho N. R. (2019). Analysis of major carotenoids and fatty acid composition of freshwater microalgae. Heliyon 5 (4), e01529. doi: 10.1016/j.heliyon.2019.e01529 31049438 PMC6484207

[B93] Song H. Lu Q. Hou L. Li M. (2021). The genes crucial to carotenoid metabolism under elevated CO2 levels in carrot (Daucus carota L.). Sci. Rep. 11 (1), 12073. doi: 10.1038/s41598-021-91522-7 34103616 PMC8187729

[B94] Sun T. Rao S. Zhou X. Li L. (2022). Plant carotenoids: recent advances and future perspectives. Mol. Horticulture 2 (1), 1–21. doi: 10.1186/s43897-022-00023-2 PMC10515021 37789426

[B95] Sun T. Yuan H. Cao H. Yazdani M. Tadmor Y. Li L. (2018). Carotenoid metabolism in plants: the role of plastids. Mol. Plant 11 (1), 58–74. doi: 10.1016/j.molp.2017.09.010 28958604

[B96] Sun T. Yuan H. Chen C. Kadirjan-Kalbach D. K. Mazourek M. Osteryoung K. W.. (2020). ORHis, a natural variant of OR, specifically interacts with plastid division factor ARC3 to regulate chromoplast number and carotenoid accumulation. Mol. Plant 13 (6), 864–878. doi: 10.1016/j.molp.2020.03.007 32222485

[B97] Vásquez-Caicedo A. L. Heller A. Neidhart S. Carle R. (2006). Chromoplast morphology and β-carotene accumulation during postharvest ripening of mango cv. ‘Tommy atkins. J. Agric. Food Chem. 54 (16), 5769–5776. doi: 10.1021/jf060747u 16881676

[B98] Wang S. Qiu Y. Zhu F. (2021). Kiwifruit (Actinidia spp.): A review of chemical diversity and biological activities. Food Chem. 350, 128469. 33485721 10.1016/j.foodchem.2020.128469

[B99] Watkins J. L. Pogson B. J. (2020). Prospects for carotenoid biofortification targeting retention and catabolism. Trends Plant Sci. 25 (5), 501–512. doi: 10.1016/j.tplants.2019.12.021 31956035

[B100] Welsch R. Zhou X. Yuan H. Álvarez D. Sun T. Schlossarek D.. (2018). Clp protease and OR directly control the proteostasis of phytoene synthase, the crucial enzyme for carotenoid biosynthesis in arabidopsis. Mol. Plant 11 (1), 149–162. doi: 10.1016/j.molp.2017.11.003 29155321

[B101] Wen X. Heller A. Wang K. Han Q. Ni Y. Carle R.. (2020). Carotenogenesis and chromoplast development during ripening of yellow, orange and red colored Physalis fruit. Planta 251 (5), 95. doi: 10.1007/s00425-020-03383-5 32274590

[B102] Wickham H. (2009). (New York, NY, USA:Elegant graphics for data analysis (ggplot Springer).

[B103] Wu C. Sun L. Lv Y. Cui H. Wang X. Gao P.. (2020). Functional characterization and in silico analysis of phytoene synthase family genes responsible for carotenoid biosynthesis in watermelon (Citrullus lanatus L.). Agron. (Basel) 10 (8), 1077. doi: 10.3390/agronomy10081077

[B104] Xia H. Zhou Y. Lin Z. Guo Y. Liu X. Wang T.. (2022). Characterization and functional validation of β-carotene hydroxylase AcBCH genes in Actinidia chinensis (Oxford University Press (OUP). 10.1093/hr/uhac063 PMC9123235 35611182

[B105] Yazdani M. Sun Z. Yuan H. Zeng S. Thannhauser T. W. Vrebalov J.. (2018). Ectopic expression of ORANGE promotes carotenoid accumulation and fruit development in tomato (Wiley). 10.1111/pbi.12945 PMC6330546 29729208

[B106] You M. K. Lee Y. J. Kim J. K. Baek S. A. Jeon Y. A. Lim S. H.. (2020). The organ-specific differential roles of rice DXS and DXR, the first two enzymes of the MEP pathway, in carotenoid metabolism in Oryza sativa leaves and seeds. BMC Plant Biol. 20 (1), 167. doi: 10.1186/s12870-020-02357-9 32293285 PMC7161295

[B107] Yu G. Wang L. G. Han Y. He Q. Y. (2012). clusterProfiler: an R package for comparing biological themes among gene clusters. Omics: J. Integr. Biol. 16 (5), 284–287. doi: 10.1089/omi.2011.0118 PMC3339379 22455463

[B108] Zeb A. Ullah F. (2017). Reversed phase HPLC-DAD profiling of carotenoids, chlorophylls and phenolic compounds in Adiantum capillus-veneris leaves. Front. Chem. 5, 29. doi: 10.3389/fchem.2017.00029 28497036 PMC5406511

[B109] Zeng Y. Du J. Wang L. Pan Z. Xu Q. Xiao S.. (2015). A comprehensive analysis of chromoplast differentiation reveals complex protein changes associated with plastoglobule biogenesis and remodeling of protein systems in sweet orange flesh. Plant Physiol. (Bethesda) 168 (4), 1648–1665. doi: 10.1104/pp.15.00645 PMC4528763 26056088

[B110] Zhang J. Guo S. Ren Y. Zhang H. Gong G. Zhou M.. (2017). High-level expression of a novel chromoplast phosphate transporter ClPHT4;2 is required for flesh color development in watermelon. New Phytol. 213 (3), 1208–1221. doi: 10.1111/nph.14257 27787901

[B111] Zhang M. Leng P. Zhang G. Li X. (2009). Cloning and functional analysis of 9-cis-epoxycarotenoid dioxygenase (NCED) genes encoding a key enzyme during abscisic acid biosynthesis from peach and grape fruits. J. Plant Physiol. 166 (12), 1241–1252. doi: 10.1016/j.jplph.2009.01.013 19307046

[B112] Zhou Q. Li Q. Li P. Zhang S. Liu C. Jin J.. (2019). Carotenoid cleavage dioxygenases: identification, expression, and evolutionary analysis of this gene family in tobacco. Int. J. Mol. Sci. 20 (22), 5796. doi: 10.3390/ijms20225796 31752180 PMC6888377

[B113] Zhou X. Welsch R. Yang Y. Álvarez D. Riediger M. Yuan H.. (2015). Arabidopsis OR proteins are the major posttranscriptional regulators of phytoene synthase in controlling carotenoid biosynthesis. Proc. Natl. Acad. Sci. - PNAS 112 (11), 3558–3563. doi: 10.1073/pnas.1420831112 25675505 PMC4371912

