# Supplementary material for: Comparative transcriptomic and plastid development analysis sheds light on the differential carotenoid accumulation in kiwifruit flesh
Source: Front Plant Sci. 2023 Aug 30;14:1213086. doi: 10.3389/fpls.2023.1213086 (PMC10499360; doi:10.3389/fpls.2023.1213086)
Supplement: Supplementary file 1 [file DataSheet_1.pdf]

Supplementary information

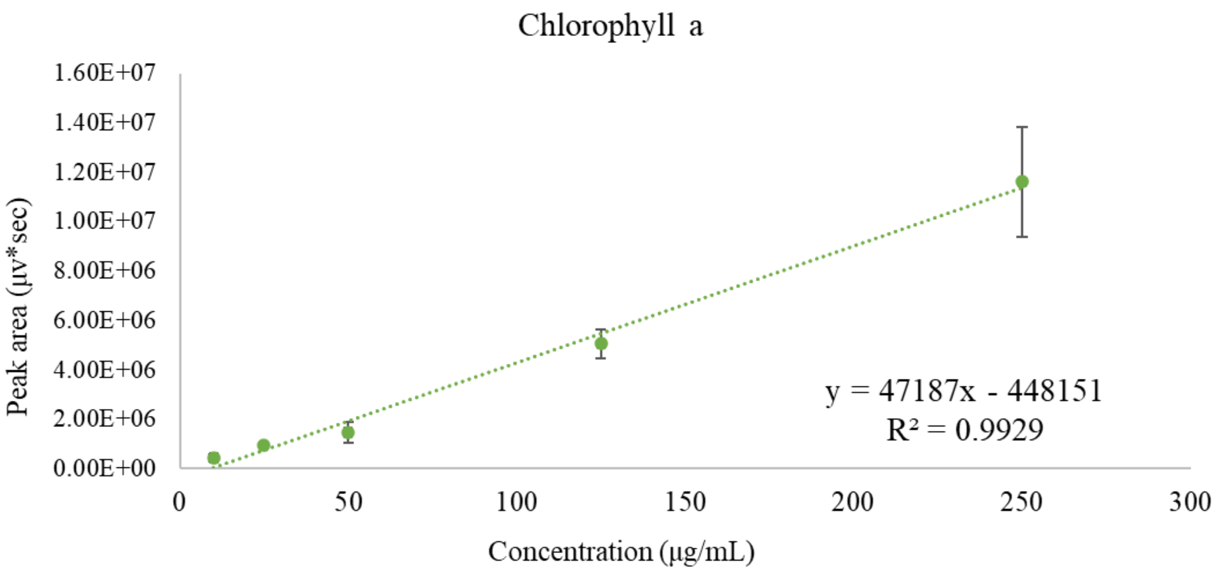

Supplementary Figure 1: Standard curve of chlorophyll a at 430nm wavelength. The curve was prepared by plotting concentration (µg/mL) of the chlorophyll standard against the peak area (µv*sec). The limit of detection (LOD) and limit of quantification for chlorophyll a were 0.001µg/mL and 0.002µg/mL respectively.

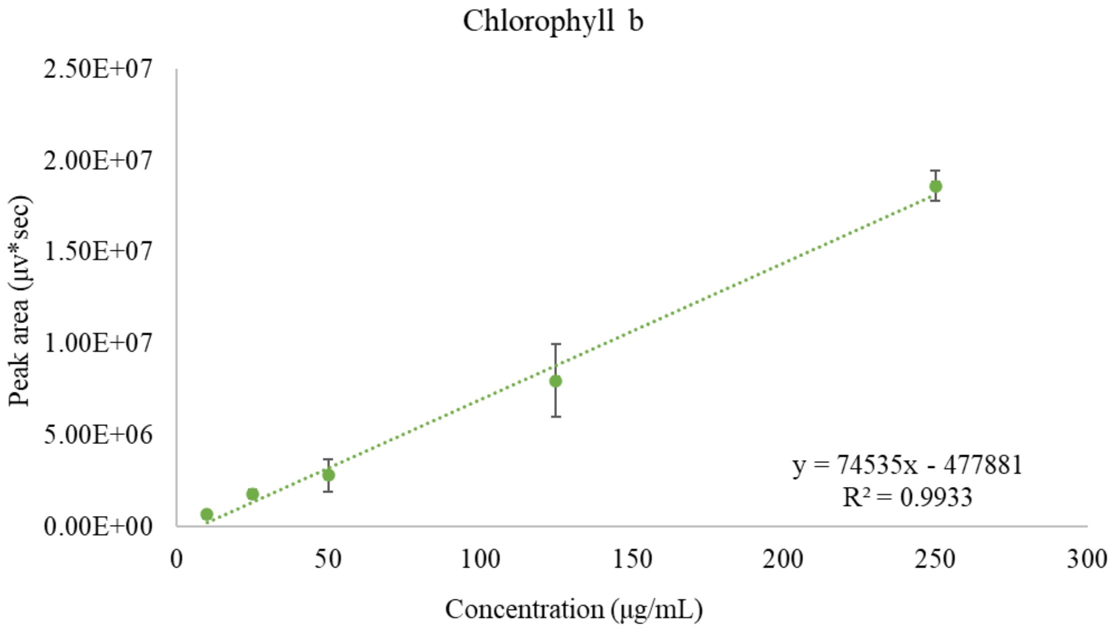

Supplementary Figure 2: Standard curve of chlorophyll b at 450nm wavelength. The curve was prepared by plotting concentration (µg/mL) of the chlorophyll standard against the peak area (µv*sec). The limit of detection (LOD) and limit of quantification for chlorophyll b were 0.0004µg/mL and 0.001µg/mL respectively.

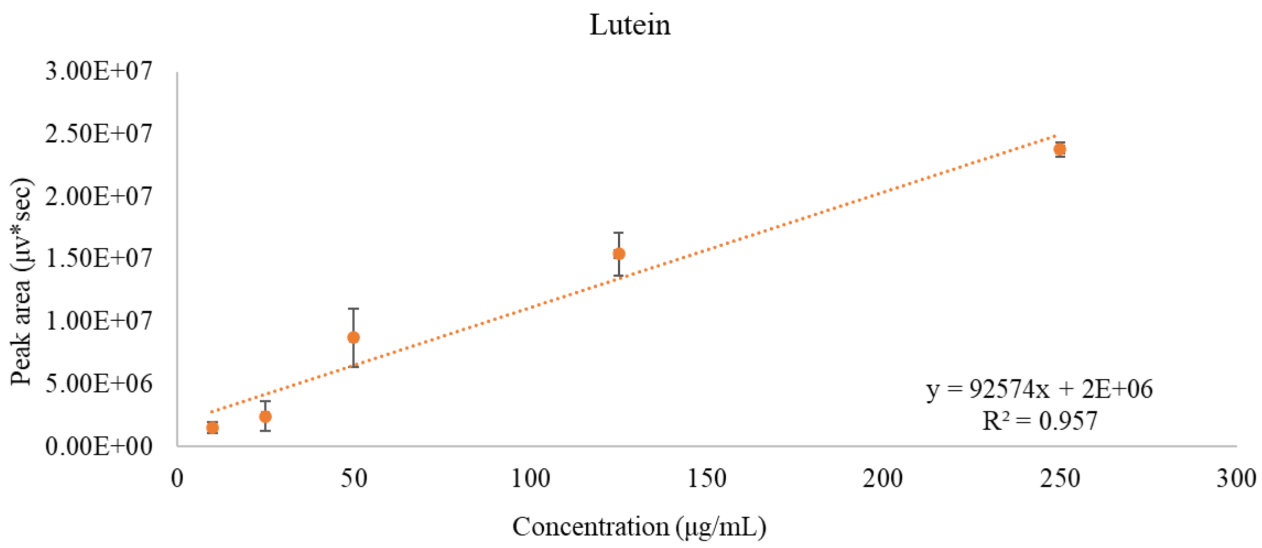

Supplementary Figure 3: Standard curve of lutein at 450nm wavelength. The curve was prepared by plotting concentration (µg/mL) of the lutein standard against the peak area (µv*sec). The limit of detection (LOD) and limit of quantification for lutein were 0.001µg/mL and 0.003µg/mL respectively.

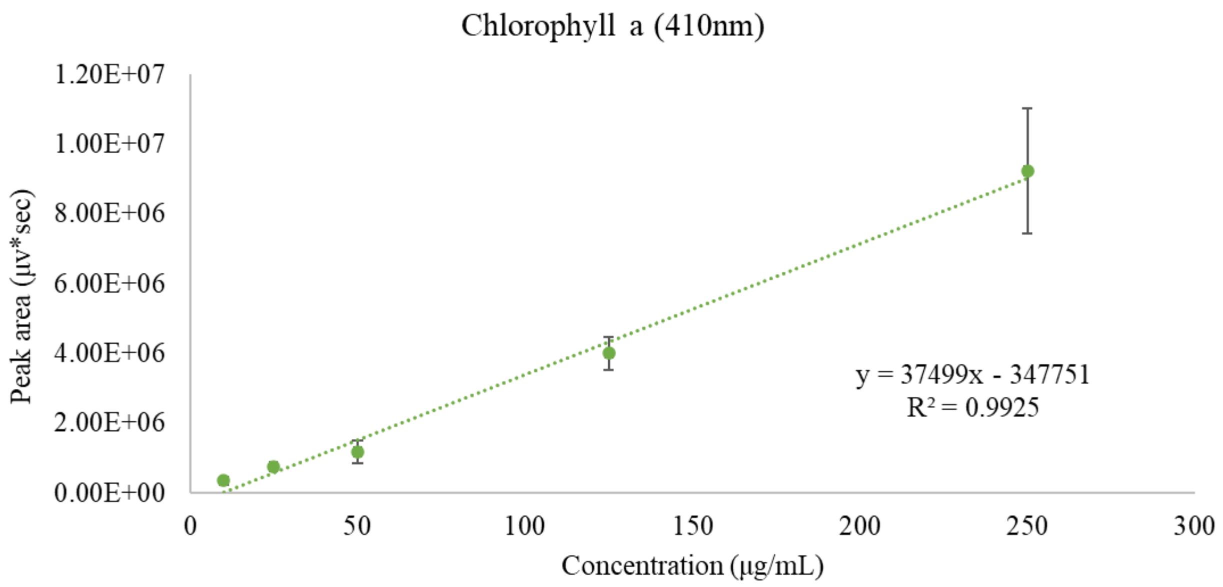

Supplementary Figure 4: Standard curve of chlorophyll a at 410nm wavelength (to quantify chlorophyll derivatives detected at 410nm). The curve was prepared by plotting concentration (µg/mL) of the chlorophyll standard against the peak area (µv*sec).

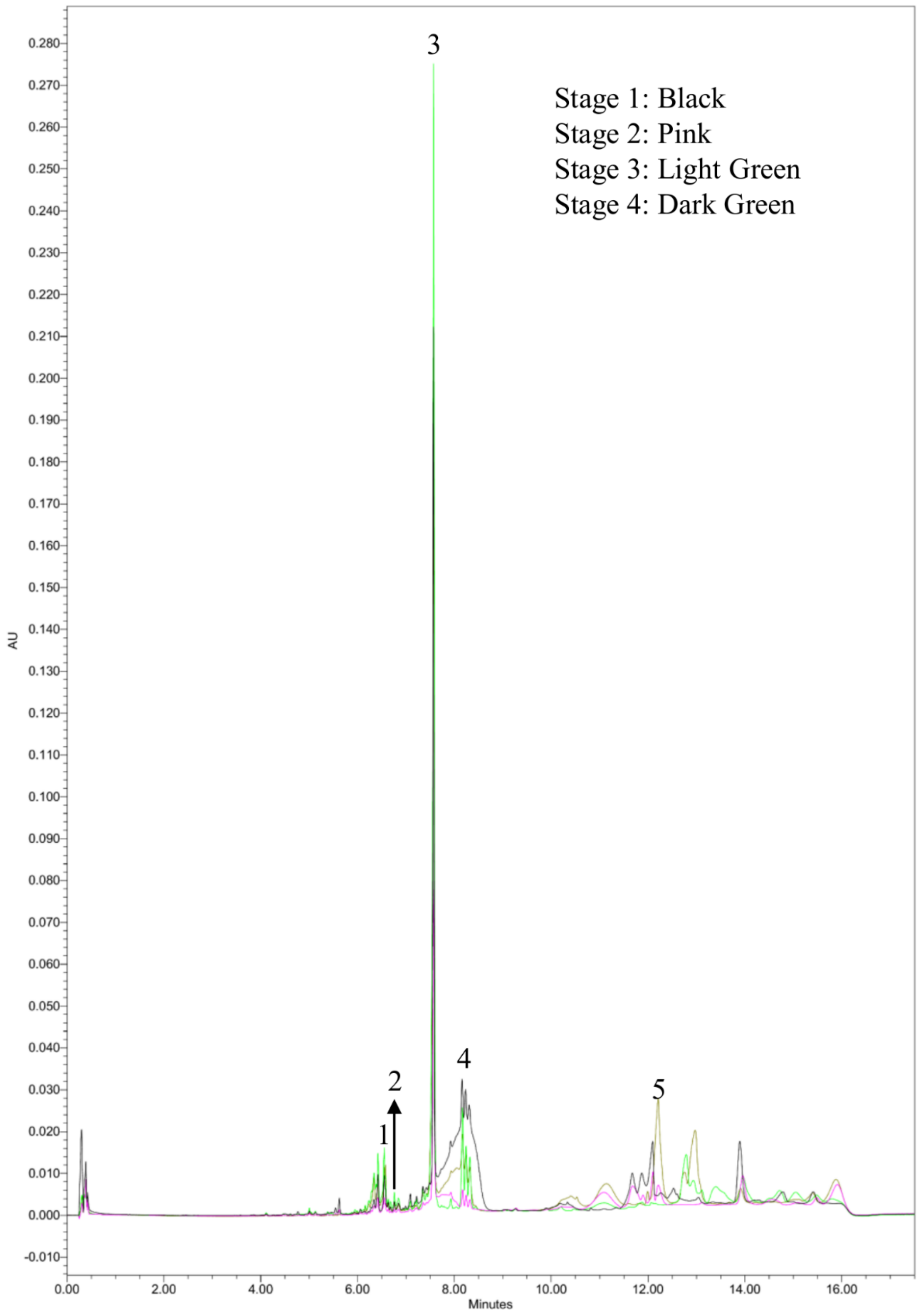

Supplementary Figure 5: Chromatogram from *A. arguta* flesh (the four ripening stages) extracted at 450nm. Carotenoids detected and quantified are labelled as (1) neoxanthin (2) violaxanthin (3) lutein (4) lutein isomers (5) chlorophyll b

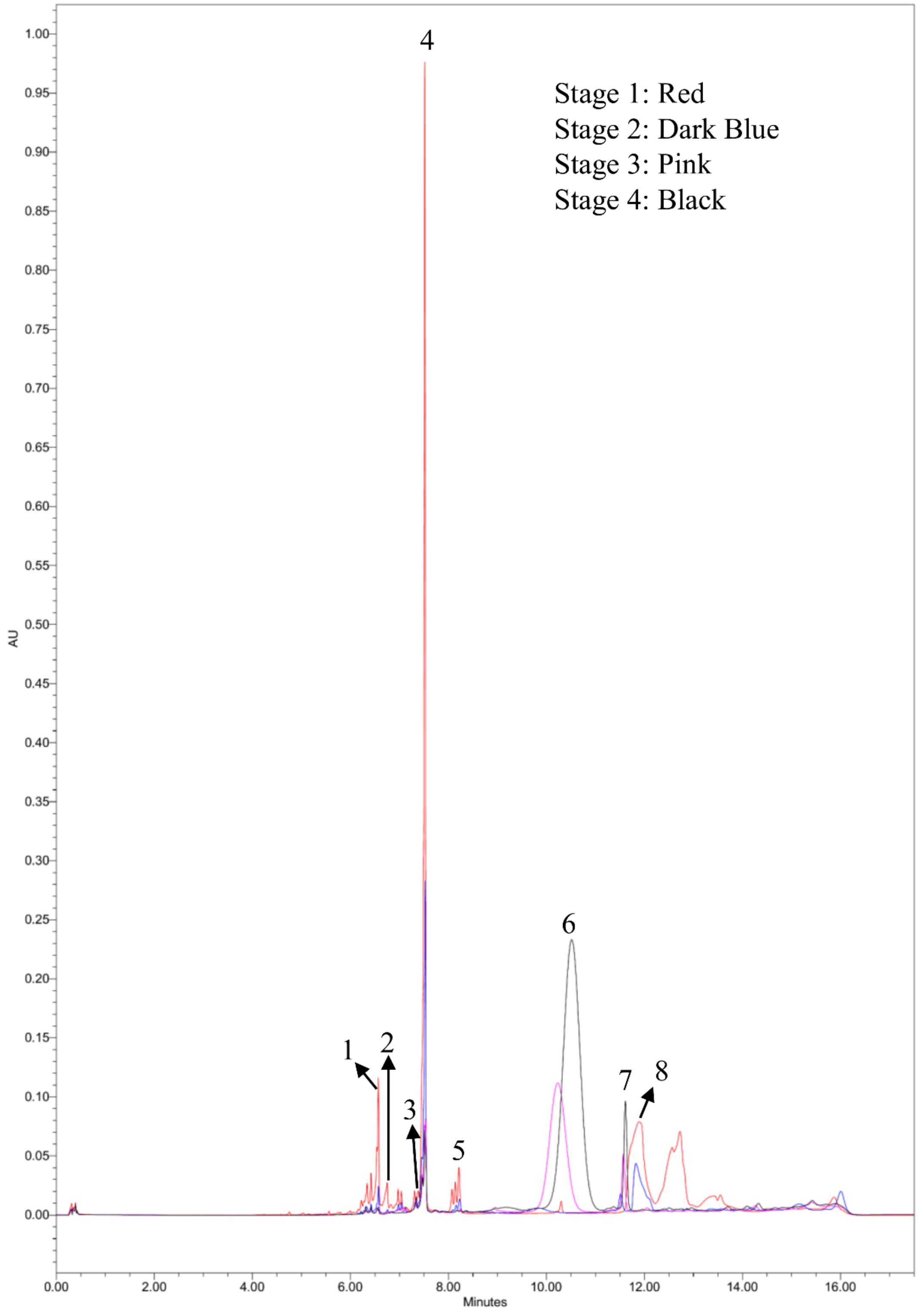

Supplementary Figure 6: Chromatogram from *A. polygama* flesh (the four ripening stages) extracted at 450nm. Carotenoids detected and quantified are labelled as (1) neoxanthin (2) violaxanthin (3) luteoxanthin (4) lutein (5) lutein isomers (6) trans-β-carotene (7) cis-β-carotene (8) chlorophyll b

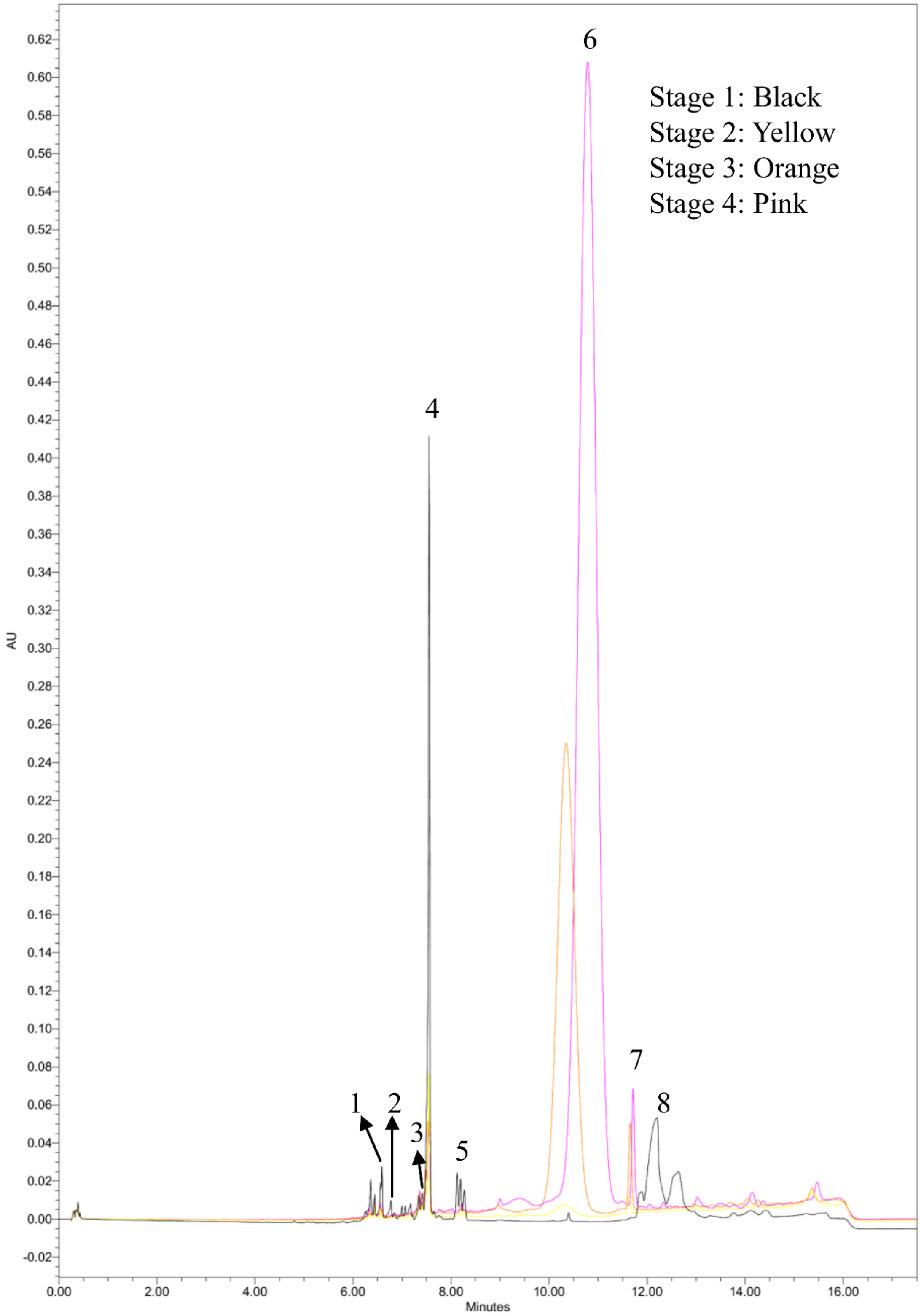

Supplementary Figure 7: Chromatogram from *A. valvata* flesh (the four ripening stages) extracted at 450nm. Carotenoids detected and quantified are labelled as (1) neoxanthin (2) violaxanthin (3) luteoxanthin (4) lutein (5) lutein isomers (6) trans-β-carotene (7) cis-β-carotene (8) chlorophyll b

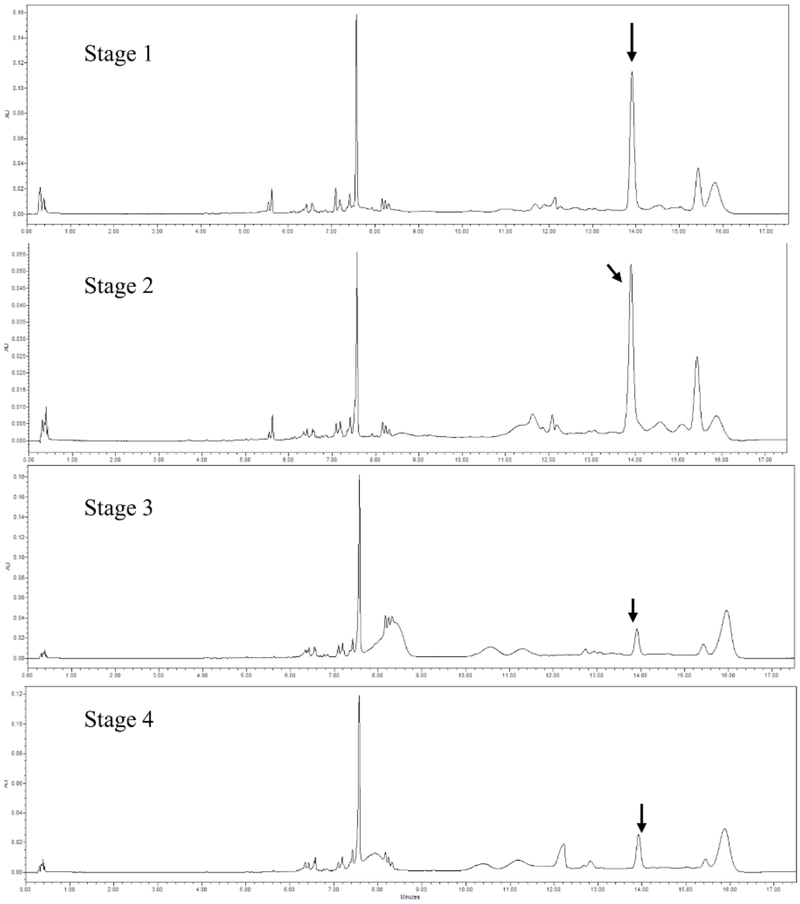

Supplementary Figure 8: Chromatogram from *A. arguta* flesh (the four ripening stages) extracted at 430nm for detecting and quantifying chlorophyll a peak (arrows)

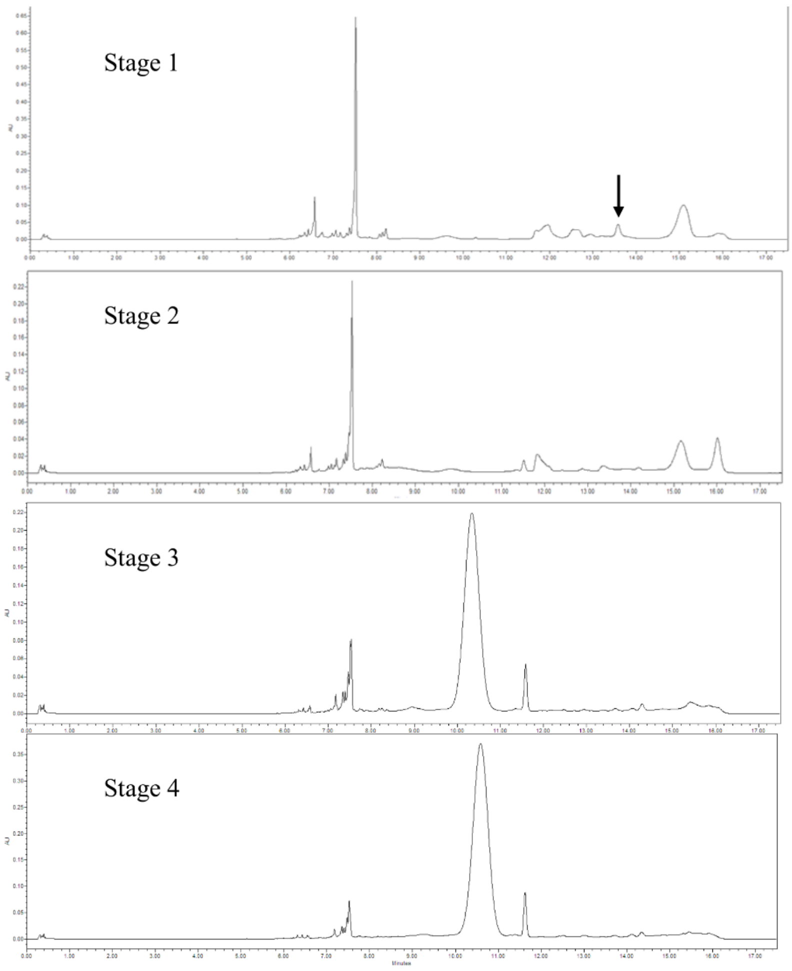

Supplementary Figure 9: Chromatogram from *A. polygama* flesh (the four ripening stages) extracted at 430nm for detecting and quantifying chlorophyll a peak (arrows)

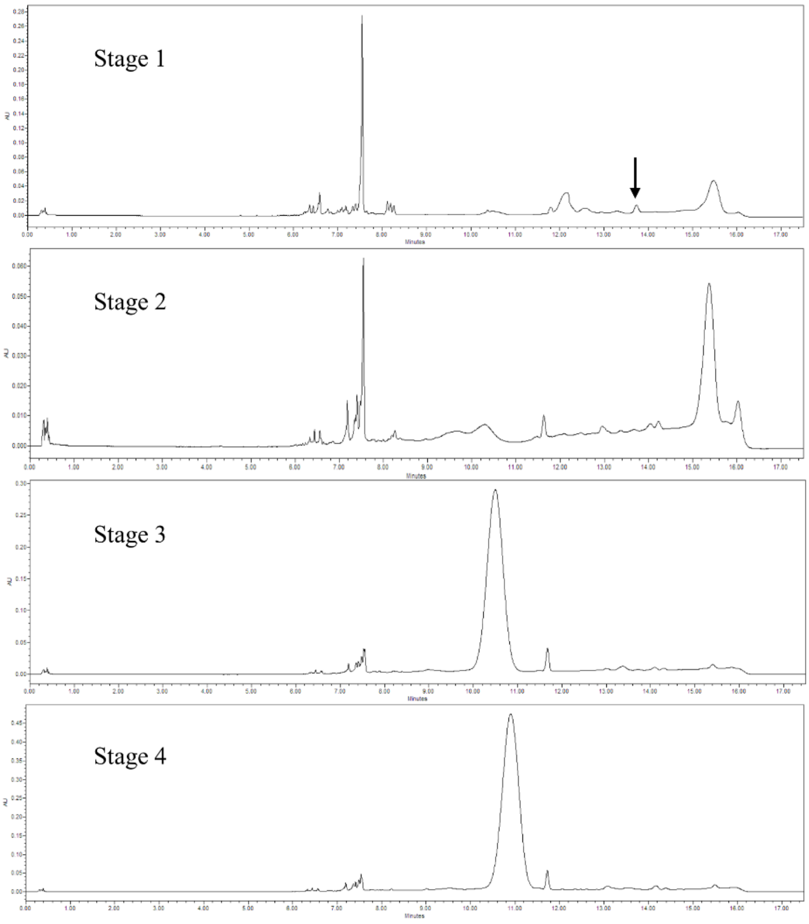

Supplementary Figure 10: Chromatogram from *A. valvata* flesh (the four ripening stages) extracted at 430nm for detecting and quantifying chlorophyll a peak (arrows)

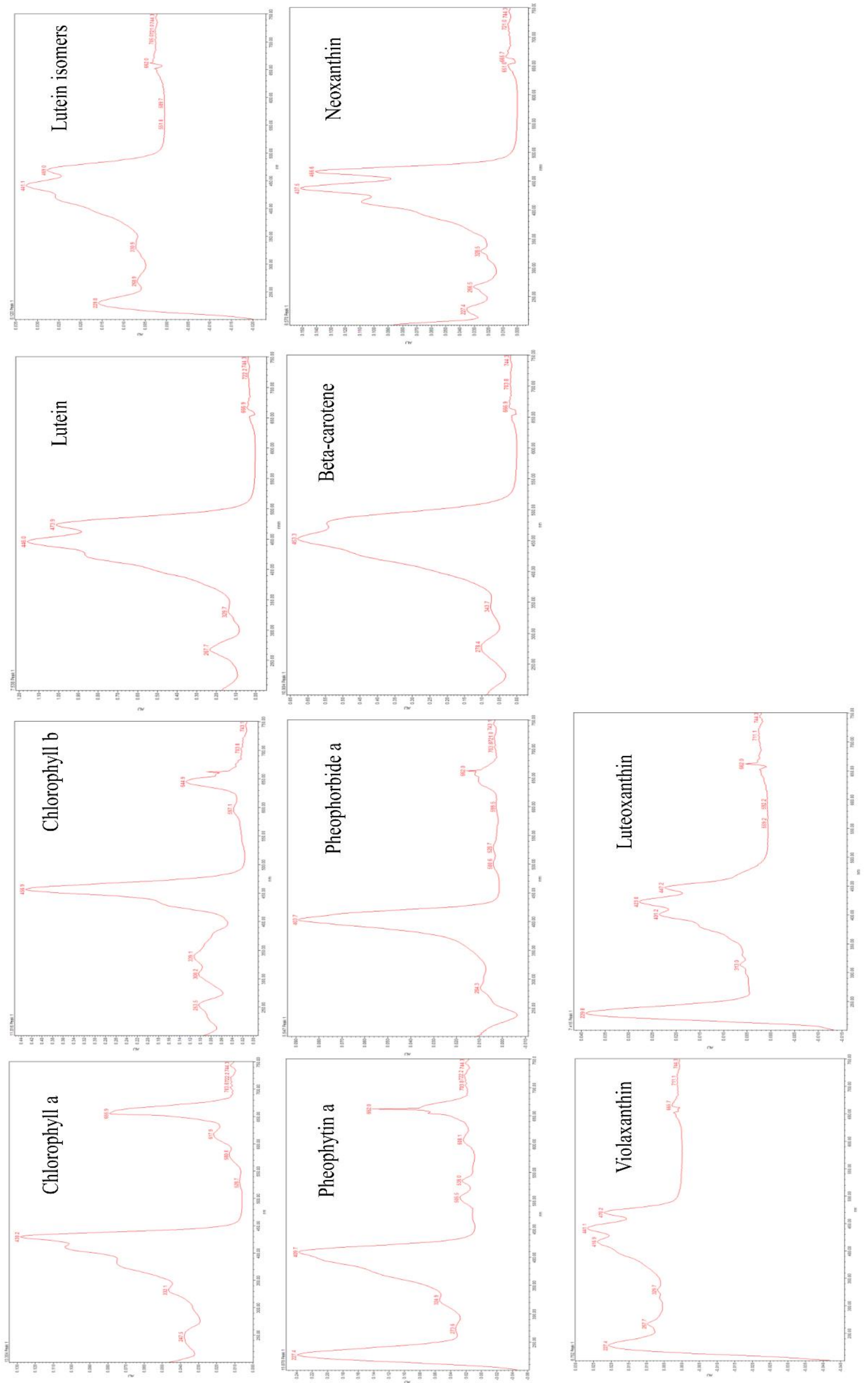

Supplementary Figure 11: UV-spectra of the identified carotenoid and chlorophyll peaks in the three *Actinidia* species

Table 1 Carotenoids and chlorophyll concentrations in flesh (during fruit ripening)

Carotenoids and chlorophylls detected	*A. arguta* (Concentration µg/g DW)				*A. polygama* (Concentration µg/g DW)				*A. valvata* (Concentration µg/g DW)			
	S1	S2	S3	S4	S1	S2	S3S	S4	S1	S2	S3	S4
Total chlorophyll a (chlorophyll a and its derivatives)	14.67 (SD=7.3)	11.89 (SD=9.9)	7.50 (SD=5.1)	8.88 (SD=3.5)	56.17 (SD=8.6)	15.94 (SD=0.7)	8.34 (SD=8.2)	2.23 (SD=1.4)	16.60 (SD=13.7)	15.61 (SD=17.4)		
Chlorophyll b	0.68 (SD=0.2)	0.37 (SD=0.08)	0.13 (SD=0.04)	0.64 (SD=0.1)	7.75 (SD=2.01)	1.24 (0.02)			3.60 (SD=0.3)			
Neoxanthin	0.04 (SD=0.02)	0.01 (SD=0.002)	0.01 (SD=0.01)	0.01 (SD= 0.003)	0.39 (SD=0.08)	0.09 (SD=0.02)	0.03 (SD=0.01)	0.03 (SD=0.004)	0.12 (SD=0.02)			
Violaxanthin	0.01 (SD=0.002)	0.005 (SD=0.003)	0.007 (SD=0.00)	0.005 (SD=0.00)	0.22 (SD=0.08)	0.01 (SD=0.006)	0.004 (SD=0.0)		0.09 (SD=0.02)	0.003 (SD=0.0)	0.008 (SD=0.001)	0.01 (SD=0.0)
Lutein	1.08 (SD=0.04)	0.43 (SD=0.1)	0.54 (SD=0.01)	0.59 (SD=0.09)	4.63 (SD=0.9)	1.23 (SD=0.04)	0.54 (SD=0.05)	0.36 (SD=0.1)	2.34 (SD=0.3)	0.45 (SD=0.03)	0.46 (SD=0.06)	0.35 (SD=0.03)
Lutein isomers	0.18 (SD=0.04)	0.07 (SD=0.02)	0.12 (SD=0.01)	0.09 (SD=0.01)	0.35 (SD=0.08)	0.11 (SD=0.007)			0.39 (SD=0.08)			
Luteoxanthin					0.11 (SD=0.02)				0.12 (SD=0.02)			
Total β-carotene						4.85 (SD=0.4)	14.61 (SD=3.7)	21.34 (SD=2.6)		12.36 (SD=0.05)	20.86 (SD=2.6)	25.88 (SD=10.63)
