# Supplementary material for: Comparative transcriptomic and plastid development analysis sheds light on the differential carotenoid accumulation in kiwifruit flesh
Source: Front Plant Sci. 2023 Aug 30;14:1213086. doi: 10.3389/fpls.2023.1213086 (PMC10499360; doi:10.3389/fpls.2023.1213086)
Supplement: Supplementary file 2 [file DataSheet_2.pdf]

**Supplementary information**

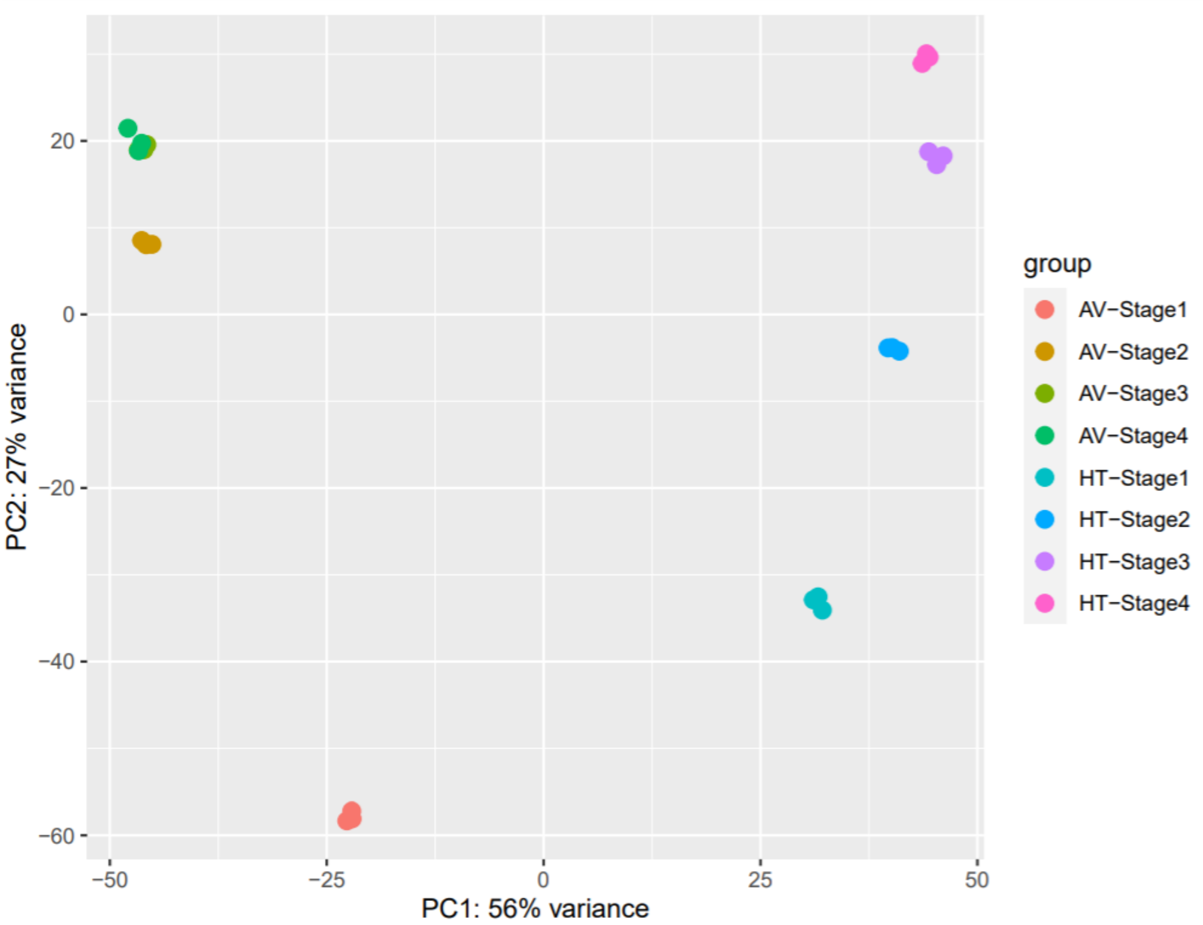


**Supplementary Figure 1:** PCA plot of RNA-Sequencing data for all the samples. A clear distinction can be observed between *Actinidia valvata* (orange) and *Actinidia arguta* (green) and the four stages of development (mature green, S1; breaker, S2; colour-change, S3; ripe, S4). Each dot represents a replicate.

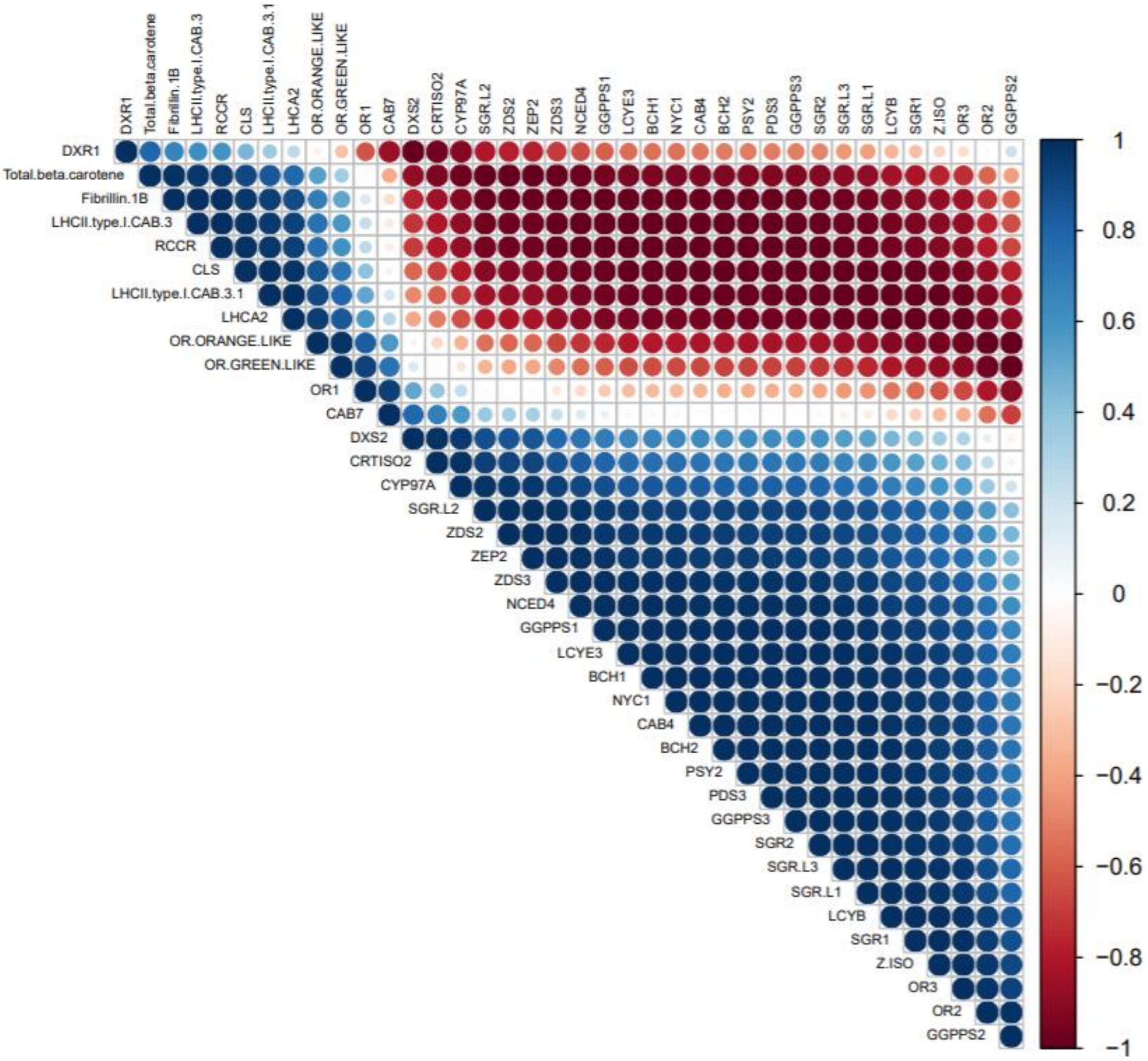


**Supplementary Figure 2:** Correlation matrix of gene transcripts and total β-carotene in *A. valvata* fruit. Values represent correlation coefficients with colour gradient with blue to red indicating strong to weak correlations.

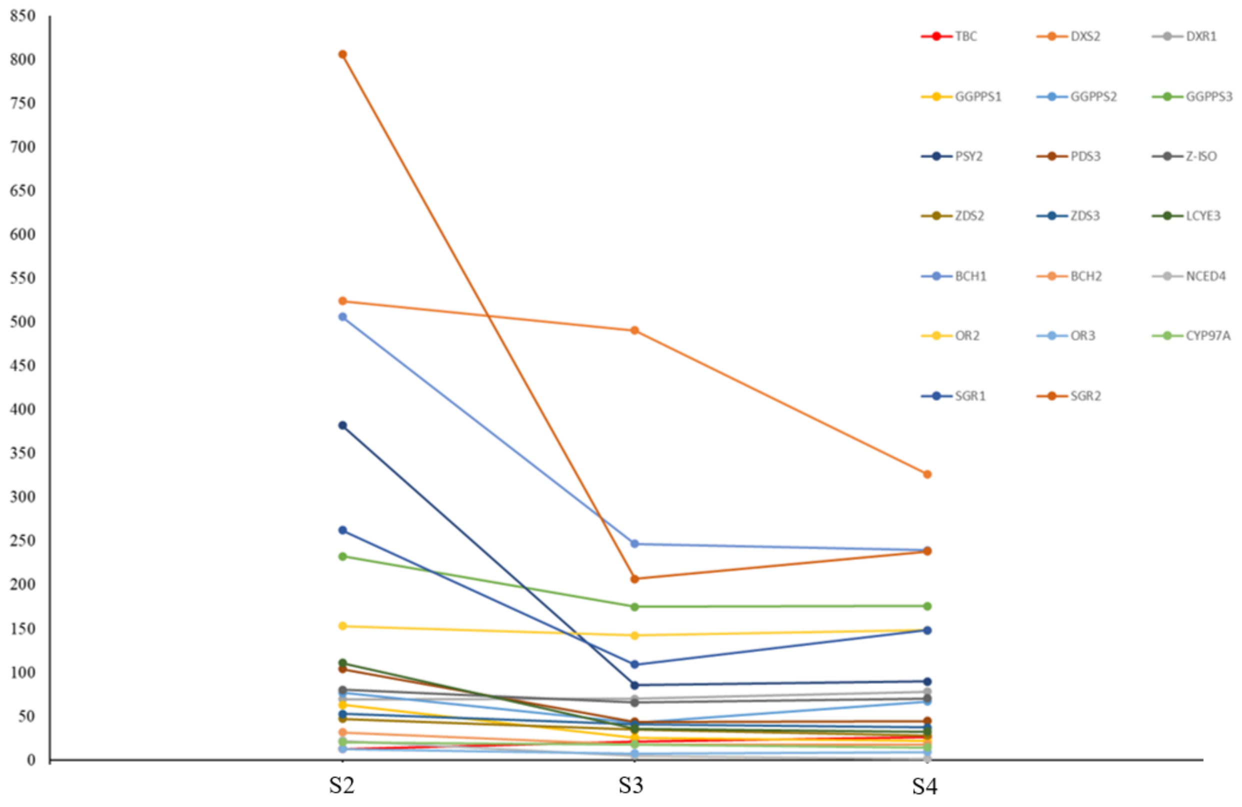


**Supplementary Figure 3:** Graph showing the differences in the accumulation pattern between carotenoid pathway gene transcripts and total β-carotene concentration in *A. valvata* fruit during ripening.

**Supplementary Table 1:** FPKM of the carotenogenesis genes listed in this study.

| **Accession number** | **Gene** | ***A. valvata*** | | | | ***A. arguta*** | | | |
|---|---|---|---|---|---|---|---|---|---|
| | | **S1** | **S2** | **S3** | **S4** | **S1** | **S2** | **S3** | **S4** |
| Acc01029.1 | DXS1 | 5.3 | 1.7 | 4.0 | 5.9 | 10.7 | 3.3 | 6.1 | 3.3 |
| Acc04331.1 | DXS2 | 50.5 | 524.3 | 490.5 | 326.5 | 52.6 | 37.8 | 42.8 | 12.2 |
| Acc05388.1 | DXS3 | 1.7 | 1.2 | 1.0 | 0.9 | 0.6 | 3.6 | 2.1 | 1.4 |
| Acc07941.1 | DXS4 | 8.2 | 3.3 | 6.5 | 5.8 | 2.8 | 6.4 | 2.1 | 1.6 |
| Acc12472.1 | DXS5 | 13.5 | 16.9 | 18.6 | 16.3 | 11.3 | 16.9 | 11.1 | 12.5 |
| Acc00743.1 | DXS6 | 10.4 | 1.2 | 13.9 | 7.2 | 4.2 | 0.9 | 155.0 | 317.6 |
| Acc18139.1 | DXR1 | 37.7 | 69.5 | 69.8 | 78.2 | 86.5 | 136.0 | 77.4 | 93.8 |
| Acc29787.1 | DXR2 | 4.3 | 10.1 | 10.0 | 11.5 | 12.0 | 8.6 | 6.1 | 6.8 |
| Acc06087.1 | GGPPS1 | 55.3 | 62.9 | 25.7 | 21.6 | 29.1 | 7.8 | 14.8 | 20.9 |
| Acc23677.1 | GGPPS2 | 107.0 | 76.9 | 42.4 | 66.9 | 153.8 | 57.5 | 52.7 | 27.4 |
| Acc31056.1 | GGPPS3 | 69.0 | 232.8 | 174.8 | 176.0 | 67.8 | 179.3 | 354.0 | 470.7 |
| Acc05332.1 | PSY1 | 12.9 | 6.4 | 2.5 | 2.0 | 26.5 | 17.8 | 7.4 | 2.5 |
| Acc24233.1 | PSY2 | 20.8 | 382.0 | 85.5 | 89.9 | 28.1 | 31.0 | 20.9 | 5.1 |
| Acc25447.1 | PSY3 | 4.3 | 38.1 | 4.0 | 5.9 | 33.2 | 51.5 | 25.2 | 11.3 |
| Acc07518.1 | PDS1 | 20.2 | 1.1 | 0.9 | 1.9 | 5.0 | 1.4 | 1.3 | 2.2 |
| Acc22366.1 | PDS2 | 14.1 | 25.8 | 13.5 | 11.1 | 28.0 | 29.2 | 12.2 | 5.2 |
| Acc27031.1 | PDS3 | 41.6 | 104.1 | 43.6 | 44.4 | 28.5 | 16.4 | 7.3 | 4.3 |
| Acc03153.1 | Z-ISO | 16.3 | 80.3 | 65.6 | 70.5 | 14.7 | 21.1 | 20.0 | 16.6 |
| Acc07147.1 | ZDS1 | 10.5 | 6.7 | 6.7 | 6.5 | 5.9 | 3.8 | 3.5 | 3.8 |
| Acc09433.1 | ZDS2 | 16.7 | 47.1 | 34.6 | 28.4 | 20.1 | 25.8 | 14.7 | 11.5 |
| Acc17205.1 | ZDS3 | 19.5 | 52.7 | 40.7 | 37.1 | 19.0 | 30.1 | 21.5 | 17.6 |
| Acc06683.1 | CRTISO1 | 2.7 | 4.0 | 4.3 | 5.0 | 2.1 | 2.2 | 1.3 | 1.1 |
| Acc13329.1 | CRTISO2 | 21.1 | 18.8 | 16.7 | 12.0 | 59.6 | 120.6 | 104.2 | 52.0 |
| Acc03946.1 | LCYB | 15.1 | 17.1 | 7.2 | 9.4 | 15.4 | 28.9 | 20.1 | 16.3 |
| Acc00929.1 | LCYE1 | 15.9 | 1.0 | 0.8 | 0.6 | 15.9 | 1.8 | 0.7 | 0.6 |
| Acc09832.1 | LCYE2 | 1.7 | 0.5 | 0.7 | 1.0 | 7.4 | 4.2 | 1.2 | 1.1 |
| Acc23239.1 | LCYE3 | 13.2 | 110.9 | 35.8 | 32.6 | 18.6 | 26.9 | 46.8 | 29.7 |
| Acc24679.1 | CYP97A | 6.4 | 20.4 | 17.7 | 14.2 | 9.6 | 19.4 | 10.2 | 9.1 |
| Acc03419.1 | BCH1 | 8.7 | 506.3 | 246.9 | 239.4 | 21.1 | 20.3 | 28.8 | 16.2 |
| Acc20244.1 | BCH2 | 4.0 | 31.5 | 17.7 | 17.9 | 3.3 | 2.2 | 3.0 | 1.5 |
| Acc09339.1 | ZEP1 | 13.9 | 0.8 | 0.8 | 0.6 | 2.0 | 0.3 | 0.2 | 0.3 |
| Acc10322.1 | ZEP2 | 7.7 | 14.5 | 10.4 | 8.3 | 14.3 | 44.4 | 20.3 | 14.6 |
| Acc15122.1 | ZEP3 | 17.8 | 1.0 | 1.3 | 1.8 | 38.3 | 6.1 | 4.4 | 0.6 |
| Acc05502.1 | VDE1 | 1.6 | 0.8 | 1.2 | 0.7 | 3.1 | 1.8 | 0.7 | 1.4 |
| Acc27545.1 | VDE2 | 11.7 | 11.4 | 13.9 | 16.7 | 18.0 | 12.9 | 5.0 | 6.3 |
| Acc28609.1 | CCD1 | 0.0 | 0.1 | 0.1 | 0.1 | 0.1 | 0.1 | 0.2 | 0.2 |

| | | | | | | | | | |
|---|---|---|---|---|---|---|---|---|---|
| Acc29073.1 | CCD2 | 2.2 | 0.1 | 0.1 | 0.2 | 0.6 | 0.1 | 0.1 | 0.0 |
| Acc29490.1 | CCD3 | 22.9 | 0.0 | 0.1 | 0.0 | 3.8 | 0.3 | 0.0 | 0.0 |
| Acc13541.1 | NCED1 | 0.4 | 0.0 | 0.0 | 0.0 | 0.1 | 0.0 | 0.0 | 0.0 |
| Acc19200.1 | NCED2 | 4.9 | 1.7 | 1.4 | 1.0 | 6.6 | 0.4 | 0.1 | 0.0 |
| Acc21143.1 | NCED3 | 0.0 | 0.1 | 0.0 | 0.4 | 0.0 | 0.0 | 0.1 | 0.4 |
| Acc26525.1 | NCED4 | 9.3 | 22.0 | 4.9 | 1.7 | 0.5 | 1.3 | 2.8 | 2.2 |
| Acc03610.1 | OR1 | 10.8 | 9.6 | 11.8 | 9.1 | 15.5 | 17.2 | 10.1 | 5.5 |
| Acc13400.1 | OR2 | 78.5 | 153.2 | 142.4 | 148.3 | 48.0 | 70.1 | 56.3 | 45.5 |
| Acc22952.1 | OR3 | 9.6 | 12.7 | 7.4 | 9.4 | 5.8 | 7.5 | 6.4 | 3.7 |
| Acc27899.1 | OR-L1 | 54.4 | 22.1 | 29.6 | 23.8 | 42.4 | 37.1 | 21.8 | 12.3 |
| Acc30794.1 | OR-L2 | 8.3 | 1.9 | 3.6 | 2.6 | 10.2 | 3.8 | 0.8 | 0.6 |
| Acc15738.1 | SGR2 | 7.4 | 805.8 | 206.9 | 238.1 | 0.0 | 0.0 | 0.1 | 0.5 |
| Acc15739.1 | SGR1 | 7.7 | 262.3 | 108.9 | 148.2 | 3.4 | 0.1 | 0.3 | 0.1 |
| Acc19958.1 | SGR-L1 | 7.6 | 2824.4 | 1054.9 | 1281.2 | 7.6 | 3.5 | 6.9 | 2.9 |
| Acc24073.1 | SGR-L2 | 0.4 | 0.8 | 0.6 | 0.5 | 1.2 | 1.5 | 1.3 | 3.3 |
| Acc28873.1 | SGR-L3 | 10.3 | 321.0 | 142.2 | 161.1 | 89.2 | 53.9 | 37.0 | 12.5 |

**Table 2** Pearson's correlation (*r*) comparing the gene expression of carotenoid pathway genes to total β-carotene concentration in *A. valvata* during fruit ripening.

| **Gene name** | **Pearson's coefficient (*r*)** | ***p*-Value** |
|---|---|---|
| *DXS2* | -0.872 | 0.011* |
| *DXR1* | 0.799 | 0.001** |
| *GGPPS1* | -0.959 | 0.210 |
| *GGPPS2* | -0.419 | 0.038* |
| *GGPPS3* | -0.923 | 0.008** |
| *PSY2* | -0.925 | 0.122 |
| *PDS3* | -0.925 | 0.101 |
| *Z-ISO* | -0.760 | 0.011* |
| *ZDS2* | -0.999 | 0.106 |
| *ZDS3* | -0.988 | 0.055 |
| *CRTISO2* | -0.933 | 0.288 |
| *LCYB* | -0.833 | 0.165 |
| *LCYE3* | -0.942 | 0.152 |
| *CYP97A* | -0.975 | 0.364 |
| *BCH1* | -0.938 | 0.038* |
| *BCH2* | -0.925 | 0.390 |
| *ZEP2* | -0.999 | 0.135 |
| *NCED4* | -0.974 | 0.211 |
| *OR1* | -0.029 | 0.071 |
| *OR2* | -0.573 | 0.001** |
| *OR3* | -0.734 | 0.097 |
| *SGR1* | -0.810 | 0.044* |
| *SGR2* | -0.912 | 0.091 |
| *SGR-L1* | -0.88 | 0.046* |
| *SGR-L2* | -0.999 | 0.021* |
| *SGR-L3* | -0.890 | 0.044* |
| *CLS* | 0.900 | 0.001** |
| *LHCA2* | 0.789 | 0.018* |

| | | |
|---|---|---|
| *LHCII type I CAB-3* | 0.964 | 0.018* |
| *LHCII type I CAB-3* | 0.843 | 0.018* |
| *CAB7* | -0.375 | 0.145 |
| *RCCR* | 0.958 | 0.035* |
| *NYC1* | -0.938 | 0.229 |
| *CAB4* | -0.930 | 0.019* |
| *FIBRILLIN 1B* | 0.981 | 0.025* |

p- Values showing statistically significant correlation; * $p < 0.05$; ** $p < 0.01$.
